# Amyloid Forming Human Lysozyme Intermediates are Stabilized by Non‐Native Amide‐π Interactions

**DOI:** 10.1002/advs.202503957

**Published:** 2025-06-25

**Authors:** Minkoo Ahn, Julian O. Streit, Christopher A. Waudby, Tomasz Włodarski, Angelo Miguel Figueiredo, John Christodoulou, Janet R. Kumita

**Affiliations:** ^1^ School of Biochemistry University of Bristol University Walk Bristol BS8 1TD UK; ^2^ Research Department of Structural and Molecular Biology Biosciences University College London Gower Street London WC1E 6BT UK; ^3^ Centre for Misfolding Diseases Yusuf Hamied Department of Chemistry University of Cambridge Lensfield Road Cambridge CB2 1EW UK; ^4^ Department of Pharmaceutical and Biological Chemistry School of Pharmacy University College London 29‐39 Brunswick Square London WC1N 1AX UK; ^5^ Institute of Biochemistry and Biophysics Polish Academy of Sciences Pawińskiego 5a Warsaw 02‐106 Poland; ^6^ Coimbra Chemistry Centre Institute of Molecular Sciences (CQC‐IMS) Department of Chemistry Faculty of Science and Technology University of Coimbra Coimbra 3004‐531 Portugal; ^7^ School of Natural Sciences Birkbeck College University of London Malet Street London WC1E 7HX UK; ^8^ Department of Pharmacology University of Cambridge Tennis Court Road Cambridge CB2 1PD UK

**Keywords:** amide‐π interactions, amyloid, CEST NMR, human lysozyme, MD simulations, protein intermediate

## Abstract

Mutational variants of human lysozyme cause a rare but fatal hereditary systemic amyloidosis by populating an intermediate state that self‐assembles into amyloid fibrils. However, this intermediate state is recalcitrant to detailed structural investigation, as it is only transiently and sparsely populated. Here, this work investigates the intermediate state of an amyloid‐forming human lysozyme variant (I59T) using CEST and CPMG RD NMR at low pH. ^15^N CEST profiles probe the thermal unfolding of the native state into the denatured ensemble and reveal a distinct intermediate state. Global fitting of ^15^N CEST and CPMG data provides kinetic and thermodynamic parameters, characterizing the intermediate state populated at 0.6%. ^1^H CEST data further confirm the presence of the intermediate state displaying unusually high or low ^1^H_N_ chemical shifts. To investigate the structural details of this intermediate state, this work uses molecular dynamics (MD) simulations, which recapitulate the experimentally observed folding pathway and free energy landscape. This work observes a high‐energy intermediate state with a locally disordered β‐domain and C‐helix, stabilized by non‐native hydrogen bonding and amide‐π interactions, accounting for its anomalous ^1^H chemical shifts. Together, these NMR and MD data provide the first direct structural information on the intermediate state, offering insights into targeting lysozyme amyloidosis.

## Graphical abstract

Mutational variants of human lysozyme cause fatal systemic amyloidosis by depositing kilograms of protein in the viscera of patients. Central to this process is a partially unfolded protein intermediate state that initiates aggregation into amyloid fibrils. Combining ^15^N/^13^C/^1^H CEST NMR and MD simulations, this work identifies and characterizes this transient intermediate state, populated only at 0.6% and stabilized by non‐native amide‐π interactions.

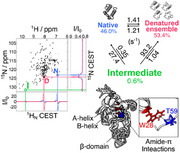

## 1 Introduction

Many globular proteins undergo locally cooperative unfolding, forming transient intermediate states that self‐assemble into amyloid fibrils associated with neurodegenerative and systemic diseases.^[^ ^1^ ^]^ Human lysozyme is a globular protein with a persistent native structure, consisting of two structural domains ‐ the α‐domain (residues 1–38 and 86–130) with four α‐helices and the β‐domain (residues 39–85) with β‐sheet structure ‐ stabilized by four disulfide bonds (C6‐C128, C30‐C116, C65‐C81 and C77‐C95). Previous studies have shown that mutations in its genetic code cause it to populate a partially unfolded intermediate state, which aggregates and accumulates in the viscera of patients, reaching up to kilogram quantities.^[^ ^2^ ^]^ It is thought that the intermediate state retains the native α‐domain structure, while the β‐domain is mostly denatured.^[^ ^3^ ^]^ The β‐sheet region of the β‐domain is of particular importance, as disease‐related mutations occur in this region,^[^ ^2^ ^]^ it is selectively denatured in the intermediate state,^[^ ^3a,c^ ^]^ and establishesinter‐molecular interactions^[^ ^4^ ^]^ to form the fibril core in amyloid.^[^ ^5^ ^]^ The disease‐related variants have a higher propensity to populate the intermediate state^[^ ^3^, ^6^ ^]^ and aggregate more rapidly than the wild‐type (WT).^[^ ^7^ ^]^

Previously, we investigated the local cooperativity in the thermal unfolding of the WT and mutational variants (I56T and I59T) of human lysozyme under acidic pH conditions.^[^ ^3c^ ^]^ Low pH conditions destabilize the native state and increase the population of the intermediate state, which is otherwise too sparsely populated to be observed under physiologically relevant conditions. Using 2D HSQC spectra and both far‐ and near‐UV CD spectra with increasing temperatures at pH 1.2, we revealed a pseudo‐two‐state unfolding mechanism, which involves a cooperative loss of native tertiary structure, followed by the progressive unfolding of the denatured ensemble.^[^ ^3c^ ^]^ The denatured ensemble ‐ a dynamic mixture of rapidly interconverting protein conformers with varying degrees of denaturation ‐ retains residual α‐domain structure,^[^ ^4^ ^]^ which is gradually lost in a non‐concerted manner as the temperature increases.^[^ ^3c^ ^]^ Such behavior is akin to the downhill folding phenomena observed in small, fast folding proteins^[^ ^8^ ^]^ and proteins that populate intermediate states capable of self‐assembling into amyloid fibrils.^[^ ^9^ ^]^

The intermediate state of human lysozyme, previously considered one of many protein conformers within the denatured ensemble, has not been structurally characterized due to the lack of first‐order cooperative unfolding and detectable heat in calorimetric analysis. However, recent advances in solution NMR spectroscopy, particularly chemical exchange saturation transfer (CEST) and CPMG relaxation dispersion (RD) NMR, have enabled the observation of protein dynamics across a broad range of timescales, providing insights into the exchange processes between protein conformers.^[^ ^10^ ^]^ CEST NMR has proven particularly useful for studying transient and sparsely populated intermediate states that are in equilibrium with native and fully unfolded protein conformers, providing accurate chemical shifts and exchange parameters for processes in second to millisecond timescales.^[^ ^11^ ^]^

To understand the unfolding process of human lysozyme and characterize the involved protein conformers, we harnessed CEST NMR at pH 1.2 to monitor the unfolding of the I59T variant, which shares key attributes with disease‐associated variants.^[^ ^12^ ^] 15^N CEST detects slow exchange between the native state and the denatured ensemble, and also an intermediate state, which is marginally populated (≈0.6%) and observed in the β‐domain and C‐helix residues. ^1^H_N_ CEST data confirmed the same exchange process and revealed unusually high or low chemical shift values for the intermediate state. Ratchet‐and‐pawl molecular dynamics (MD)^[^ ^13^ ^]^ simulations using two different force fields accurately recapitulate the folding free energy landscape of lysozyme, and reveal a high‐energy local minimum corresponding to a transient intermediate state with a compact global structure but with locally disordered β‐domain and C‐helix. Extensive unbiased simulations of these intermediate state structures reveal non‐native hydrogen bonding and amide‐π interactions as the molecular basis for the anomalous ^1^H_N_ chemical shifts from the CEST data.

Collectively, our NMR and MD simulations enable the first direct observation of the lowly populated lysozyme intermediate state and reveal how these disease‐causing structures are stabilized by non‐native interactions, providing a potential structural target for perturbing lysozyme fibril formation in systemic amyloidosis.

## 2 Results

### 2.1 The Invisible Denatured Ensemble Observed by ^15^N CEST during Thermal Unfolding

**Figure 1:**
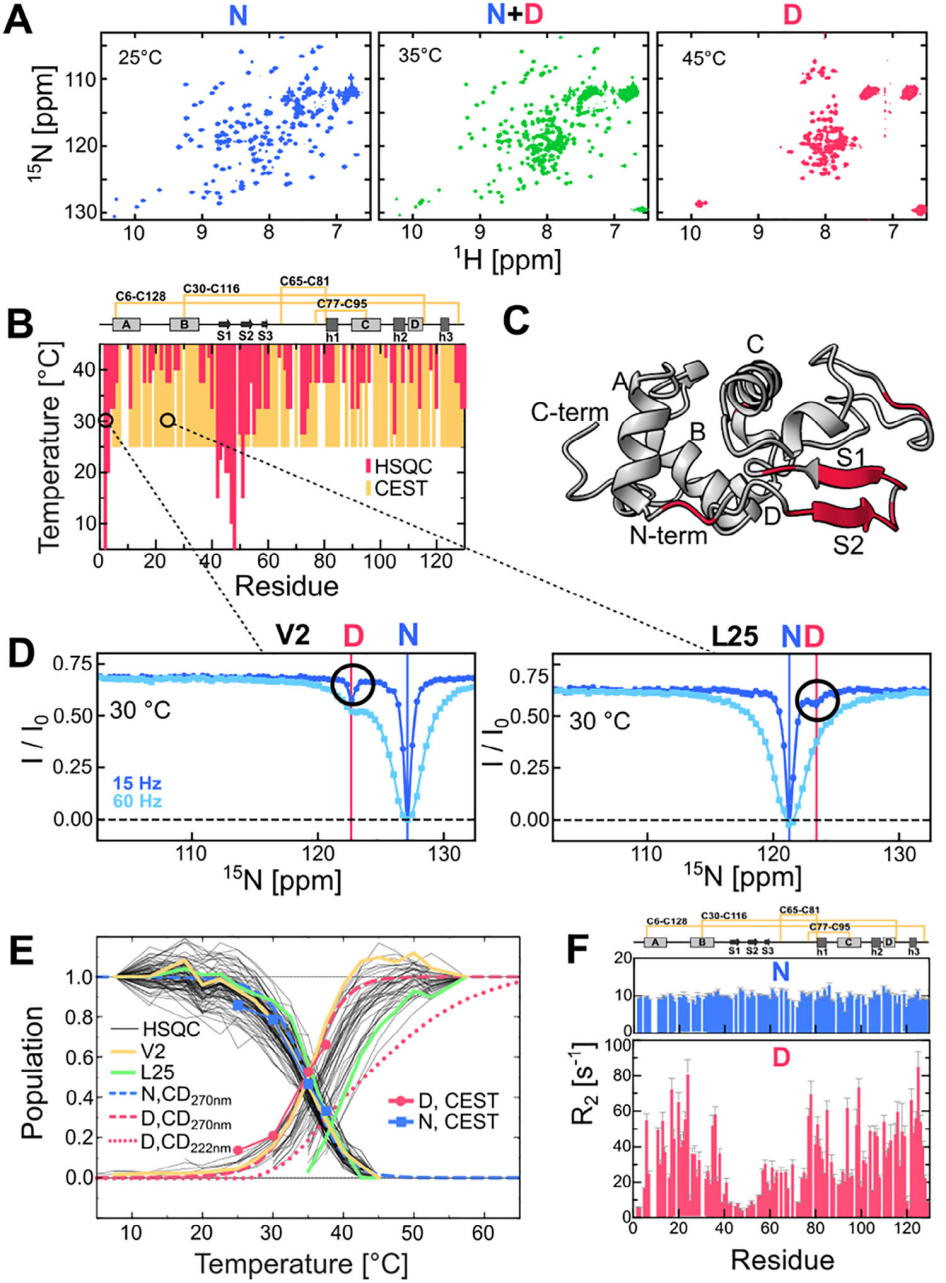
The denatured ensemble state observed by CEST NMR. A) ^1^H‐^15^N HSQC spectra of I59T at pH 1.2 at three different temperatures (25, 35 and 45 °C). B) Observation of the denatured state using ^1^H‐^15^N HSQC (5–45 °C, red) and ^15^N CEST NMR (25–45 °C, yellow). C) Residues showing denatured peaks below 30 °C, highlighted on the I59T structure (PDB ID: 2MEH^[^ ^15^ ^]^) D) ^15^N CEST profile for V2 and L25 at 30 °C. E) Thermal unfolding of I59T monitored by NMR and CD spectroscopy. Peak volumes in the HSQC spectra are shown in black solid lines, with data from V2 and L25 in yellow and green, respectively. Thermal unfolding by near‐UV CD (270 nm) was fit to a two‐state unfolding model, with native (N, blue dashed) and denatured ensemble (D, red dashed) populations indicated. Far‐UV CD (222 nm) thermal unfolding data were normalized, and the denatured population is shown as a red dotted line. ^15^N CEST data from all observed peaks (109 residues) were globally fit to a two‐state unfolding model, with N (blue squares with blue solid line) and D (red circles with red solid line) populations shown. F) ^15^N transverse relaxation rates (*R*_2_) of the N (blue) and D (red) states derived from ^15^N CEST fitting at 35 °C.

The thermal unfolding of I59T at pH 1.2 was previously monitored by recording ^1^H‐^15^N HSQC spectra and revealed a pseudo‐two state unfolding process where the native state (N) exchanges with the denatured ensemble (D), which progressively unfolds its secondary structure as the temperature increases.^[^ ^3c^ ^]^ I59T predominantly populates the N state at 25 °C and the D state at 45 °C, while at 35 °C, close to its melting temperature (*T*_m_) for tertiary structure (determined by near‐UV CD spectroscopy, Figure S1A, Supporting Information),^[^ ^3c^ ^]^ both N and D peaks are observed (**Figure** **1**A; Figure S1B, Supporting Information). As the temperature increases, different regions of the protein gradually show denatured peaks, with the N‐terminal short β‐strand (residues 2–4) and the three β‐strands in the β‐domain (residues 40–55) predominantly unfolding at low temperatures (<30 °C, Figure 1B,C,E).

To further investigate the equilibrium between the native and denatured states of lysozyme during thermal unfolding, we performed ^15^N CEST NMR experiments, which probe exchange on the second to millisecond timescales.^[^ ^14^ ^]^ Figure 1D shows the CEST profiles for two residues (V2 and L25) at 30 °C. V2, which displays its D peak at the lowest temperature (from 5 °C), shows clear N‐D state exchange at 30 °C (Figure 1D). Similarly, L25, located in one of the α‐helices (helix B), shows N‐D exchange at 30 °C, even though its D peak is only visible at higher temperatures (>35 °C) in the HSQC spectra. At 25 °C, all visible N state peaks (109 out of 118) in the 2D spectra exhibit exchange with the D state in the CEST profile (Figure 1B), except for residues with the same chemical shifts for both N and D states, preventing direct detection of exchange. This suggests that even at low temperatures, the invisible protein conformers of the D state, which are in equilibrium with the N state, can be observed using CEST, enabling us to explore the detailed exchange processes during thermal unfolding.

The CEST profiles of 109 residues were globally fit to a two‐state model, revealing the populations of both the N and D states consistent with near‐UV CD results (Figure 1E; Figure S1A, Supporting Information).^[^ ^3c^ ^]^ Figure 1F shows the *R*_2_ values of the N and D states derived from CEST data fitting. The N state exhibits uniform *R*_2_ values of ≈10 s^−1^ across the protein sequence, indicating a stable protein structure. In contrast, the D state displays variable *R*_2_ values: residues that unfold at lower temperatures (N‐terminal residues 2–4 and β‐strands residues 40–55) have lower *R*_2_ values (<10 s^−1^), indicating fast dynamics. The rest of the protein, particularly the α‐domain, shows higher *R*_2_ values, reflecting additional rapid exchange processes among protein conformers^[^ ^11a^ ^]^ with varying degrees of α‐domain denaturation. The ^15^N *R*_2_ values of the D state follow a similar trend to experimentally measured ^1^H_N_ *R*_2_ values,^[^ ^4^ ^]^ indicating consistent D state dynamics under these conditions and explaining the lower D state populations observed in the α‐domain peaks of the HSQC spectra, as exemplified by L25 (Figure 1E).

### 2.2 The Invisible Intermediate State Observed by ^15^N CEST and CPMG RD

**Figure 2:**
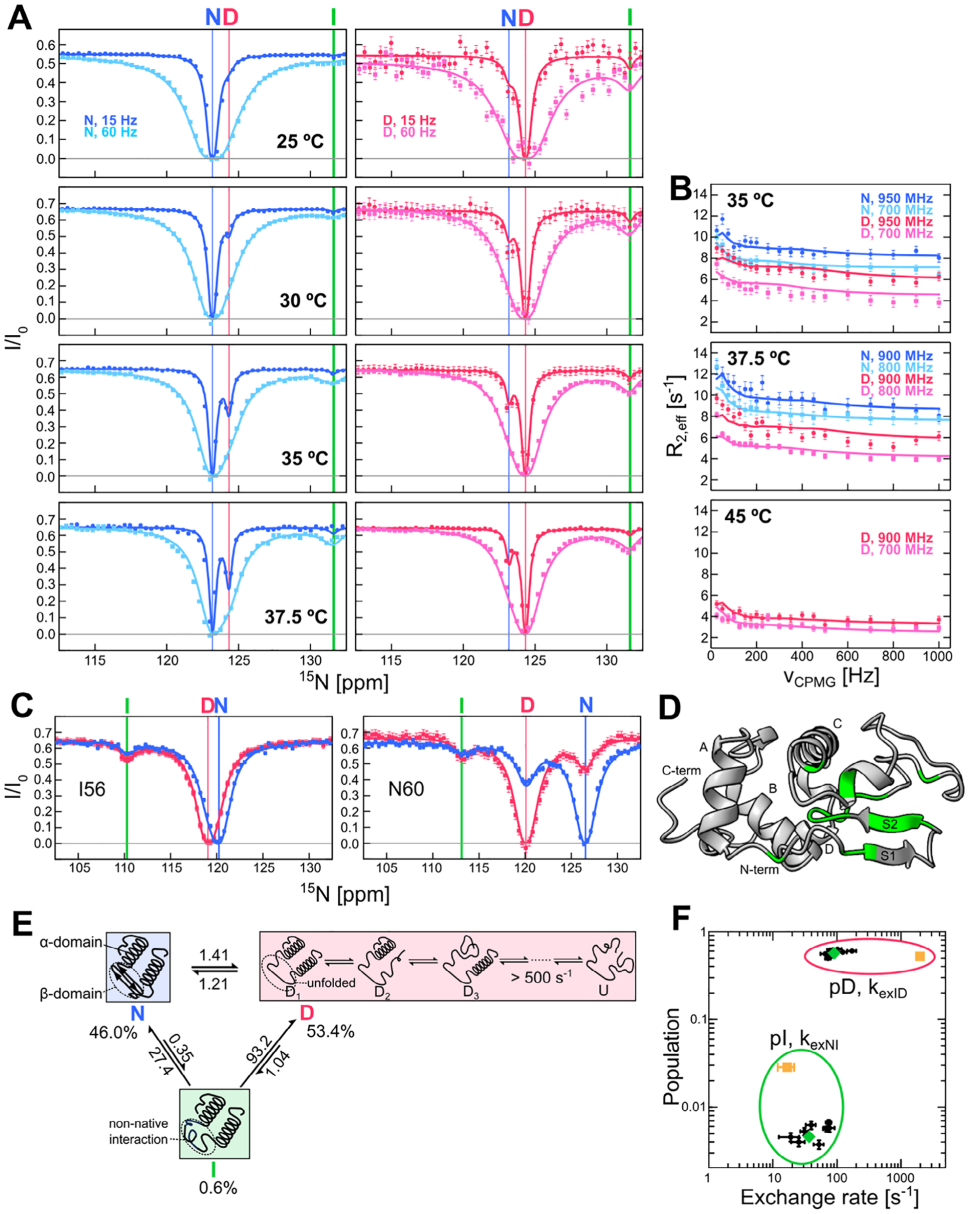
The intermediate state observed by ^15^N CEST and CPMG RD NMR. A) ^15^N CEST and B) CPMG RD data for A42 during thermal unfolding (25–45 °C). The chemical shifts of the native (N), denatured ensemble (D) and intermediate (I) states are shown in blue, red and green vertical lines, respectively. C) CEST profiles of I56 and N60 at B1 = 60 Hz. The presence of the I state is indicated by an additional CEST dip, marked by green vertical lines. D) Residues showing the I state CEST dips distinct from the D state, highlighted on the I59T structure (PDB ID:2MEH^[^ ^15^ ^]^). E) A three‐state thermal unfolding model used for the global fitting of the CEST and CPMG data from 14 residues in D. The kinetic parameters are derived from data at 35 °C. N, I and D represent the native, intermediate and denatured states based on the β‐domain and C‐helix structures. D1, D2, D3 and U represent rapidly exchanging protein conformers that make up the D state, with the U indicating a fully unfolded protein. F) Comparison of populations and exchange rates from the global fitting of the ^15^N CEST and CPMG RD data from individual β‐domain residues (black dots), all β‐domain and C‐helix residues together (green diamond) and α‐helix (helix B) residues (orange square). Data points in the red and green ellipses represent the population of the D state (with *k*_ex_ID_) and the I state (with *k*_ex_NI_), respectively.

To quantitatively investigate the thermal unfolding and associated exchange processes, we analyzed the ^15^N CEST data collected at 25–45 °C, which encompass the melting temperatures of both tertiary (35 °C) and secondary (45 °C) structures.^[^ ^3c^ ^]^ **Figure** **2**A shows the CEST profiles of A42, a residue on the S1 strand of the β‐domain. The CEST profiles from the N and D peaks recorded at two different B1 fields (15 and 60 Hz) reveal a temperature‐dependent increase in the D state population and a corresponding decrease in the N state population, shown in the changing depth of the minor state CEST dips. Additionally, a distinct CEST dip is observed at a ^15^N shift of 131.4 ppm, indicating a third state in exchange with both the N and D states. This additional state, not an artifact from H/D exchange,^[^ ^16^ ^]^ was observed in the CEST profiles of 14 residues, located in the β‐domain (A42, T43, S51, T52, D53, G55, I56, F57, Q58, T59, N60, D67, L84) and the C‐helix (A92) (Figure 2C,D; Figure S2, Supporting Information). These β‐domain and the C‐helix residues are known to be locally denatured in the intermediate state populated during unfolding^[^ ^3a,b^ ^]^ and form the core of amyloid fibrils.^[^ ^5^ ^]^ Moreover, most disease‐related point mutations occur in this region.^[^ ^2^ ^]^ These findings suggest that the third state uniquely observed in the CEST profiles is likely to be the unfolding intermediate (I) state that self‐assembles into amyloid fibrils. Additionally, V2 at the N‐terminus, which populates its D state at a very low temperature like the β‐strand residues (Figure 1B), also shows the I state, indicating the role of the N‐terminal short β‐strand (residue 2–4) in N state stability and unfolding.^[^ ^17^ ^]^ The I state is observed from all CEST profiles recorded at different field strengths (500, 700 and 950 MHz) probing consistent exchange processes (Figure S3, Supporting Information).

To further characterize the exchange processes observed by CEST, we performed ^15^N CPMG RD experiments at multiple field strengths (700, 800, 900 and 950 MHz), which probe faster millisecond exchanges.^[^ ^10^ ^]^ (Figure 2B; Figure S3, Supporting Information). The CPMG RD data from both the N and D peaks of the 14 residues at the N‐terminus, β‐domain and C‐helix show exchange processes that were quenched at high CPMG frequencies (ν_CPMG_), revealing the presence of the additional state(s) in millisecond exchange with the N and D states (Figure 2B; Figure S3, Supporting Information). We globally fitted all CEST and CPMG data from various temperatures and field strengths for each residue showing distinct I state in the CEST profiles to three‐state sequential exchange models (N ↔ I ↔ D, N ↔ D ↔ I and I ↔ N ↔ D), as well as a triangular model that includes all possible exchange processes (Figure 2E; Figure S4, Supporting Information). The triangular model provided the best fit with the lowest reduced χ^2^ BIC and AIC values (Figure S4, Supporting Information), suggesting that the I state is not an off pathway intermediate in the unfolding and folding processes. Fitting the data for each β‐domain residue resulted in consistent populations and exchange rates, and the global fitting of all these residues together yielded the same kinetic parameters (Figure 2F). These results suggest that the I state is populated through the local cooperative unfolding of the β‐domain and the C‐helix, only transiently and marginally during the thermal unfolding process with a maximum population of 0.6% (35–37.5 °C), which is near the lower detection limit of both CEST and CPMG RD experiments.^[^ ^10^ ^]^ The exchange rates (*k*_ex_) between N and I, and I and D are 27.7 and 94.3 s^−1^ at 35 °C, with free energy differences (ΔΔG) of 2.7 and −3.06 kcal mol^−1^, respectively.

Unlike the β‐domain and C‐helix residues, the rest of the α‐helix residues in the α‐domain show no indication of the I state in their CEST profiles, with D state peaks only appearing at higher temperatures (Figure 1B). The progressive thermal unfolding of α‐helices leads to the formation of a rapidly interconverting ensemble of protein conformers with varying degrees of denaturation,^[^ ^3c^ ^]^ resembling the downhill folding phenomenon observed in small, fast folding proteins with non‐concerted unfolding.^[^ ^8^, ^9^, ^18^ ^]^ As a result, α‐domain residues exhibit distinct CEST and CPMG profiles compared to the β‐domain and C‐helix residues. Their D state minor CEST dips are broader than that of the β‐domain and C‐helix residues (Figure 1F; Figure S5A, Supporting Information) and their N state CPMG peaks show smaller *R*_ex_ values (<2 s^−1^, Figure S5B, Supporting Information), both indicating faster exchange between interconverting protein conformers.^[^ ^14^ ^]^ The absence of I state CEST dips and the limited number of D state peaks for α‐domain residues make fitting their data into a three‐state unfolding model challenging, resulting in noticeably different kinetic parameters (Figure 2F). We then attempted to fit the α‐domain data with the β‐domain data into the three‐state model using either the N or D state chemical shifts for the I state. The results showed better fitting when the I state chemical shifts were assigned to the N state, suggesting that the I state retains a native‐like α‐domain structure, consistent with the predicted I state structure.^[^ ^3c^ ^]^ The *R*_2,0_ values from the CPMG analysis of denatured peaks suggest the presence of faster exchange in α‐domain residues (Figure S5C, Supporting Information). We recorded rotating frame (*R*_1ρ_) relaxation dispersion (RD) for an α‐domain residue at the C‐terminus (G129), and the global fitting of both on‐ and off‐resonance *R*_1ρ_ RD data reveal a faster exchange process between these protein conformers (Figure S5D, Supporting Information). These NMR experiments collectively reveal fast (>500 s^−1^) exchange processes between protein conformers with varying degrees of α‐domain denaturation (Figure 2E).

As previously reported, I59T exhibits reduced global cooperativity of unfolding in comparison to the WT protein, making this variant more aggregation‐prone.^[^ ^7^ ^]^ We performed CEST experiments on the WT protein at multiple temperatures (25, 35 and 45 °C) (Figure S6, Supporting Information), and found no evidence of the I state. This is likely due to the lower population of the I state in the WT,^[^ ^3^, ^7^ ^]^ which falls below the detection limit of CEST experiments.

### 2.3 ^1^H Chemical Shifts of the Intermediate State

To further investigate the intermediate (I) state of I59T observed in ^15^N CEST and CPMG experiments, we performed CEST experiments on other NMR‐observable nuclei including ^13^C and ^1^H. Both backbone (H_N_, Cα/Hα) and side chain nuclei (Cγ/Hγ, and Cδ/Hδ) CEST data revealed exchange between the N and D states, detecting the invisible D state peaks at lower temperatures (Figure S7, Supporting Information) like the ^15^N CEST results (Figure 1B). However, no intermediate state was observed in the ^13^C CEST, potentially due to the inherently low sensitivity of methyl CEST.^[^ ^14^ ^]^

**Figure 3:**
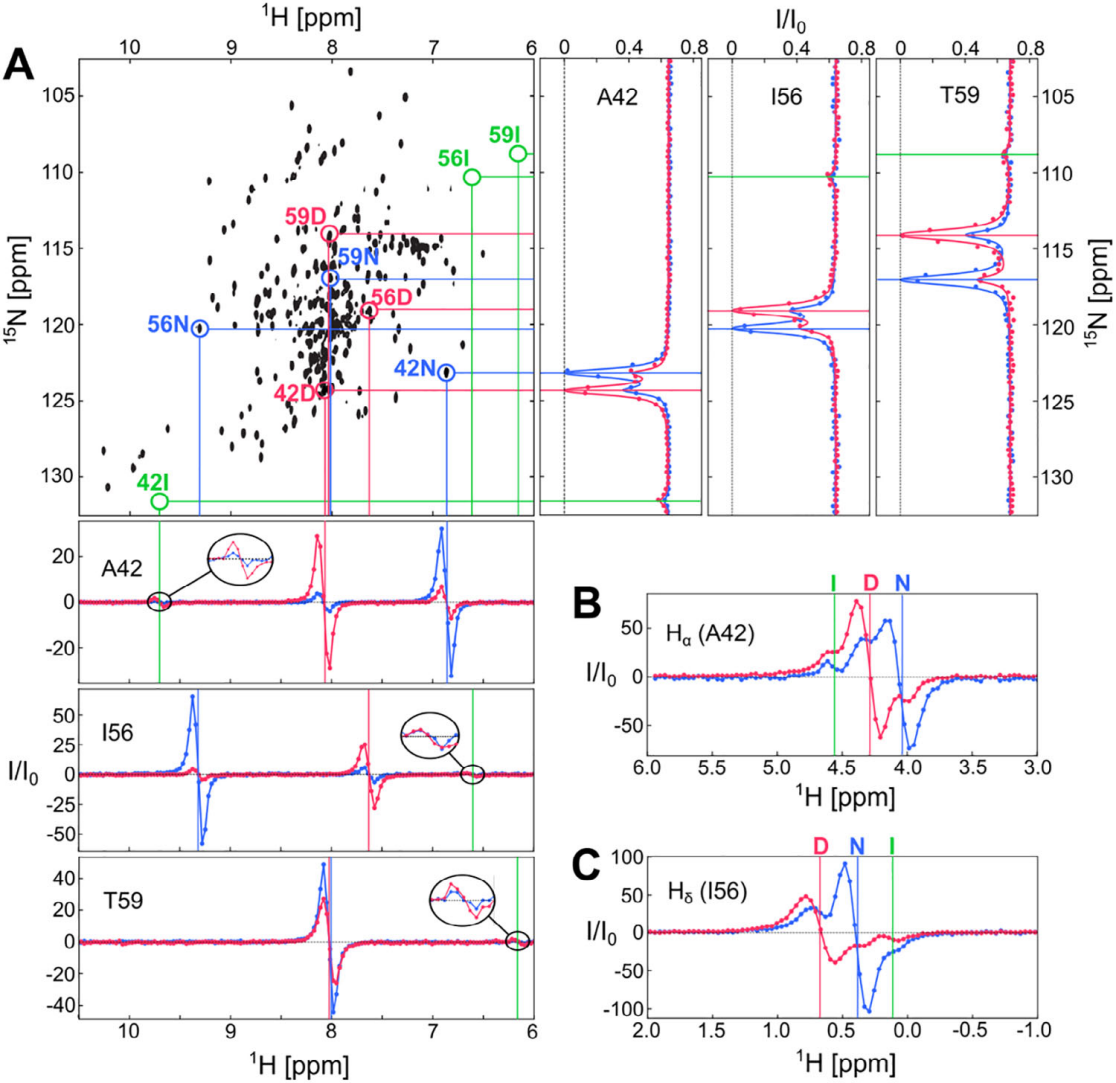
The I state monitored by ^1^H CEST. A) ^1^H_N_ and ^15^N CEST data for A42, I56 and T59 shown with the ^1^H‐^15^N HSQC spectrum at 35 °C. ^1^H_N_ CEST were recorded at a ^1^H frequency of 950 MHz. Blue and red dots connected by lines represent the native (N) and denatured (D) peaks, respectively. Green circles indicate the I state in exchange with both N and D. B) Hα CEST of A42 and C) Hδ CEST of I56 both revealing the presence of the I state.

In contrast, the ^1^H CEST data revealed both invisible D and I states. **Figure** **3**A shows the HSQC spectrum of I59T at 35 °C with ^15^N and ^1^H CEST profiles of three β‐domain residues (A42, I56 and T59). The anti‐phase absorptive ^1^H_N_ CEST profiles clearly show the I state in these β‐domain residues in exchange with both N and D states (Figure 3A). Interestingly, the I state exhibits significant chemical shift differences from both the N and D states in the ^1^H and ^15^N CEST data, indicating strong shielding (I56, I59) or deshielding (A42) effects on the observed nuclei. The I state was also detected in the ^1^H CEST profiles of Hα and Hδ nuclei of A42 and I56 (Figure 3B,C), further confirming its presence.

### 2.4 MD Simulations Reveal a Compact Intermediate State with Unstructured β‐Domain and C‐Helix

**Figure 4:**
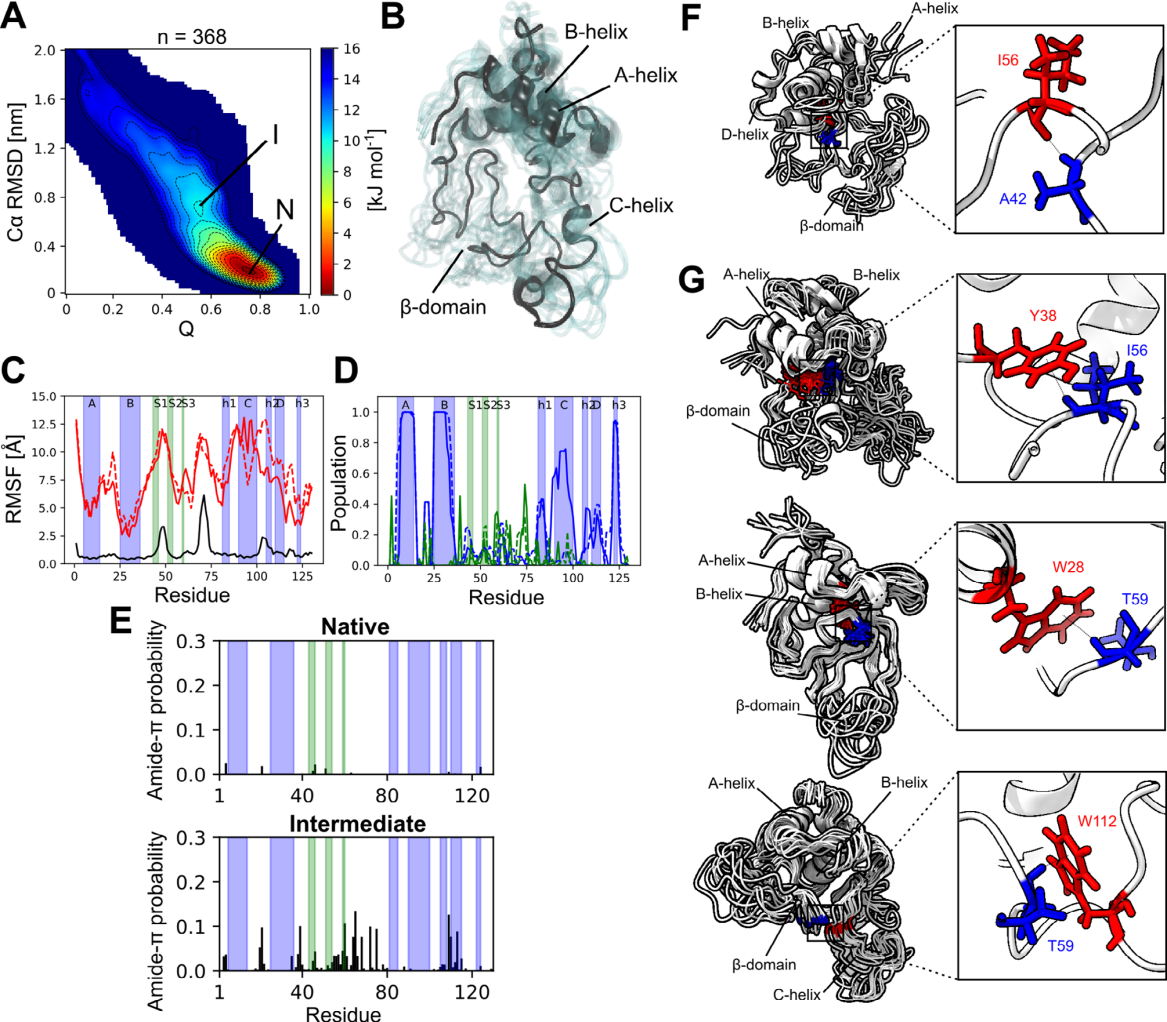
Characterization of the kinetic folding free energy landscape and intermediate state by molecular dynamics simulations. A) Kinetic folding free energy landscape of I59T calculated from all (*n* = 368) rMD trajectories that reach the native state with the a99sb‐disp force field. The regions of the native state and metastable intermediate are annotated. B) Representative ensemble of the folding intermediate state (I) from panel A. This ensemble represents the most populated cluster from the I state region in panel A. C) Backbone dynamics of the native (black) and intermediate state (red) calculated from the unbiased MD simulations for the a99sb‐disp force field, quantified by the RMSF (Cα atoms). The solid and dashed red lines represent the RMSF calculated from two halves of the ensemble (each from 4 × 10 µs of unbiased MD simulations for a different intermediate starting structure to assess the reproducibility of the structural ensembles). D) Average secondary structure populations (α‐helix ‐ blue, β‐sheet ‐ green) of the intermediate state ensemble obtained with a99sb‐disp (solid and dashed lines represent the two half ensembles). E) Ensemble‐averaged probability of forming amide‐π interactions in the native and intermediate state ensemble obtained with a99sb‐disp. F‐G) Non‐native interactions observed in the β‐domain rationalizing the intermediate state chemical shifts. F) The amide of residue A42 donates both mainchain and sidechain hydrogen bonds. The ensemble and interaction shown highlight a backbone hydrogen bond donated to I56. G) The I56 amide group participates in amide‐π hydrogen bonding and an example sub‐ensemble and interaction with Y38 are shown. The amide bond is oriented perpendicular to the ring plane. The T59 amide group also participates in amide‐π hydrogen bonding. An example ensemble and interaction with W28 are shown (sampled in a C36m simulation), as well as with W112 (sampled in an a99sb‐disp simulation).

Next, we aimed to investigate the structure of the intermediate state and sampled the folding free energy landscape of the I59T using all‐atom MD simulations in explicit solvent. Protein folding simulations are challenging due to the long timescales involved and complex folding free energy landscape, thus we took the advantage of an enhanced sampling approach referred to as adiabatic MD or ratchet‐and‐pawl MD simulations (rMD, Methods).^[^ ^13^, ^19^ ^]^ In this method, we apply a biasing force when the protein backtracks along a pre‐defined collective variable that captures folding progression (defined by, i.e., the number of established native contacts). This approach is computationally efficient and has been successfully applied to various proteins in implicit and explicit solvent.^[^ ^13^, ^19^ ^]^ Starting from 50 completely unfolded structures (with a fraction of native contacts below 0.1) with all four disulfide bonds oxidized (confirmed to be intact by the Cβ chemical shift of the Cys residues; Table S1, Supporting Information), we generated a total of 1800 trial folding trajectories (50 × 36) for two different force fields, C36m^[^ ^20^ ^]^ and a99sb‐disp,^[^ ^21^ ^]^ of which 415 and 368 reached the native state in 5 ns of biased simulation time, respectively. We then calculated the kinetic folding free energy landscape as a function of two collective variables, the Cα RMSD to the native state and Q (the fraction of native contacts) for each force field (**Figure** **4**A; Figure S8A, Supporting Information for C36m, Supporting Information). These energy landscapes contain a deep free energy minimum corresponding to the native state, and additionally a high‐energy local minimum consistent with a transient, on‐pathway kinetic folding intermediate. To identify the folding intermediate structures, we clustered conformations obtained by rMD, which belong to the intermediate state energy minimum. The most populated cluster (a99sb‐disp) represents a state with an unfolded β‐domain and a partially unfolded C‐helix (Figure 4B), consistent with previous experimental studies^[^ ^3c^ ^]^ and our ^15^N CEST experiments. A similar state is observed when clustering the intermediate state region obtained with the C36m force field (Figure S8A,B, Supporting Information).

The average folding pathway observed in the rMD simulations qualitatively recapitulates the observed folding pathway from biophysical experiments (Methods), demonstrating the delayed folding of β‐sheet and C‐helix in comparison to the fast‐folding of remaining α‐helices (Figure S8C–E, Supporting Information). This is consistent with the intermediate state obtained from clustering, which lacks native β‐sheet and C‐helix structures. An analysis of the solvent accessibility of the indole amide (W109 and W112) and side chains of Trp residues (W28, W64, W109 and W112) also describe the same folding pathway (Figure S8F–H, Supporting Information) observed by stopped‐flow CD and NMR hydrogen‐deuterium exchange experiments,^[^ ^22^ ^]^ demonstrating the delayed folding of the C‐helix (Methods). Overall, our rMD simulations predict the folding pathway from experimental studies with helices A‐C forming secondary structure rapidly, followed by helix D and then the β‐sheet. All structural elements except the C‐helix form tertiary contacts early, while the C‐helix folds late against the β‐sheet and preformed α‐domain. The intermediate state is also consistent with the I59T mutation as this increases its population by destabilizing the native state, since the sidechain of residue 59 is located between the β‐sheet and helices h2/C.

Two representative structures from the intermediate clusters with an unfolded C‐helix and β‐sheet were selected and subjected to long‐timescale unbiased MD simulations to explore the conformational space of the intermediate state further and assess its stability. We obtained 4 × 10 µs of MD data per input structure (160 µs total across both force fields) and ran unbiased simulations of the native state as an additional control (4 × 2.5 µs per force field, 20 µs total). The native state remained highly stable with average Cα‐RMSDs to the crystal structure of 1.88 ± 0.28 Å, while the intermediate state remained compact with only small increases in the radius of gyration (Figure S9A, Supporting Information). Most of the α‐domain (A, B and h3 helices) showed native‐like stable structures, while the β‐domain and C‐helix, exhibit much higher RMSF values, indicative of their increased structural dynamics in the intermediate state (Figure 4C; Figure S9B, Supporting Information). Likewise, the N‐terminal five residues also show a large increase in RMSF, consistent with NMR relaxation measurements indicating increased dynamics of the N‐terminus^[^ ^4^ ^]^ and their involvement in the population of the I state in our ^15^N CEST data (Figure 2D). Also, the analysis of secondary structure stability in the unbiased MD simulations show that the I state remained stable on the timescale of tens of microseconds in unbiased MD simulations in both force fields (Figure 4D; Figure S9C, Movies S1–S6, Supporting Information). While the intermediate state remains relatively compacted due to the lack of complete unfolding, the protein becomes significantly more exposed to the solvent relative to the native state (Figure S9D, Supporting Information).

Last, we analyzed the differences in intra‐chain contacts between the native and intermediate states and observed some common trends in both force fields (Figure S9E–G, Supporting Information). Within the β‐sheet, there is a loss of native contacts while new non‐native interactions appear. The β‐domain also loses its native contacts with helix h1 and the N‐terminus, rationalizing their increased dynamics. Additionally, the β‐domain forms non‐native interactions with helices C and h2, consistent with the observed compactness of the intermediate state and the lack of complete unfolding. While contacts between helices A and B remain largely intact, contacts between helix A and C as well as B and h2 are lost. Helix C also loses native contacts with the nearby h1 helix, which is compensated for by non‐native interactions between these structural elements. In summary, the unbiased MD simulations showed that the intermediate state identified by rMD is stable on the timescales explored here (Movies S1–S6, Supporting Information), consistent with the ensemble corresponding to metastable intermediate state.

### 2.5 Non‐Native Interactions Stabilize the Intermediate State and Elicit Anomalous ^1^H Chemical Shifts

We analyzed the intermediate state structures further to understand the structural basis of the observed chemical shifts of the I state from CEST NMR experiments. Highly acidic condition for the NMR experiments (pH 1.2) poses limits for back‐calculation of the chemical shifts^[^ ^23^ ^]^ resulting in average errors higher than that of the forward model even for the native state MD ensemble (Figure S10A, Supporting Information); however, both native and intermediate ensembles showed better agreement with their respective experimental dataset (Figure S10B, Supporting Information). Globally, the agreement with the intermediate statechemical shifts is worse compared to the native ensembles, and this appears to be dominated by the deviations in the amide proton and nitrogen chemical shifts of residues A42, I56, F57, and T59, all located in the β‐domain (Figure S10C, Supporting Information).

To characterize the intermediate state structure even further, especially the role of the non‐native interactions, we focused on residues A42, I56 and T59 that show both observable ^1^H_N_ and ^15^N chemical shifts of theintermediate state. Residue A42 has an ^1^H_N_ chemical shift of 6.90 and 9.70 ppm in the native and intermediate state, respectively, exhibiting significantly higher deshielding in the intermediate state (Figure 3A). Qualitatively, this increase in chemical shift from the native state is predicted correctly by the MD simulations, but with a smaller magnitude (Figure S10D, Supporting Information). Residue I56, on the other hand, shows a significantly lower chemical shift for the intermediate state (6.61 ppm) compared to its native state (9.37 ppm), indicating a strong shielding effect to the observed proton. Again, MD simulations predicted a lower chemical shift in the intermediate state with some structures showing chemical shifts below 7 ppm, consistent with the experimental data (Figure S10D, Supporting Information). A similar trend is seen for T59 with a native chemical shift of 8.06 ppm and intermediate shift of 6.14 ppm, suggesting a dramatic change in the average chemical environment. Our simulations correctly predict a decrease in chemical shift but with a smaller magnitude than observed experimentally (Figure S10D, Supporting Information). However, predicted chemical shifts below 7 ppm are sampled by both force fields, particularly for a99sb‐disp where some structures have predicted T59 proton shifts as low as 4 ppm. Overall, despite the large deviations from the experimental shifts, our simulations sample structures that are in qualitative agreement with the experimental data, suggesting these snapshots can provide insights into the subtle interactions sampled by these residues in the intermediate state.

We inspected the MD‐derived structures with the most deshielded A42 chemical shifts (proton chemical shift >9.5 ppm) and found that the A42 amide participates in backbone and sidechain hydrogen bonding in these structures. In simulations with the C36m force field, we found the intermediate state to predominantly donate a backbone hydrogen bond to I56 and S51 (Figure 4F), while with a99sb‐disp we mostly observed a sidechain hydrogen bond to residue N39. Thus, A42 may be involved in non‐native hydrogen bonding which could rationalize its deshielded chemical shift. For residue I56, we inspected structures with a predicted proton chemical shift of less than 6 ppm. We found that these structures exclusively show the amide of I56 in close contact with aromatic rings, oriented perpendicularly to the rings. This is consistent with amide‐π hydrogen bonding (Figure 4G), which has been observed for some well‐ordered proteins and can lead to extremely low amide proton chemical shifts (≈3 ppm).^[^ ^24^ ^]^ In the C36m simulations, the most frequent amide‐π contact involved the sidechain of W28 (0.8%) and in a99sb‐disp, a more frequent contact with Y38 was observed in ≈7.2% of simulation frames. These contacts are non‐native and are not sampled in the native state. Last, T59 exhibits an even lower chemical shift in the intermediate state, and we find that simulations sample structures with a proton chemical shift of less than 5.5 ppm. Again, consistent with amide‐π hydrogen bonding, these structures show T59 exclusively involved in close contacts with aromatic rings perpendicular to the ring plane (Figure 4G). In C36m simulations the most frequent contact involved W28 (1.8%), while W112 was the most frequently contacted sidechain by the T59 (5.7%) amide in a99sb‐disp simulations (Figure 4G).

While these amide groups are observed to interact with different parts of the protein depending on the force field (and certainly limited by sampling), we analyzed the total residue‐specific probability of amide‐π hydrogen bonding in the native and intermediate state and found that generally tryptophan sidechains followed by tyrosine, are the most common residue types to participate in this interaction. Furthermore, these interactions occur more frequently in the intermediate compared to the native state for both force fields (Figure 4E, Figure S9H, Supporting Information). Most amide‐π interactions involve amides located in the β‐domain and to a lesser extent the region of helices h2 and D (Figure 4E). These regions overlap with the regions that partially unfold from their native position and can form non‐native interactions with the partially exposed hydrophobic core, rendering the intermediate state rather compact and likely contributing to its stability. Together, the experiments and simulations therefore suggest that the unfolded regions sample non‐native interactions with a partially exposed hydrophobic core.

## 3 Discussion

The intermediate state of human lysozyme has long been elusive to biophysical analyzes despite its direct link to hereditary systemic amyloidosis. Using an acidic conditions to reduce the global cooperativity of lysozyme unfolding we previously described a pseudo‐two state model revealing the exchange between the native (N) state and denatured ensemble (D).^[^ ^3c^ ^]^ The D state comprises rapidly interconverting protein conformers with varying degrees of secondary structure denaturation. This was confirmed by the offset between unfolding curves observed via far and near‐UV CD spectroscopy, alongside the absence of latent heat in calorimetric measurements. The progressive unfolding of secondary structural elements was further supported by the gradual appearance of the cross peaks in 2D HSQC spectra with increasing temperatures, but direct information on the intermediate state has remained unavailable.

In this study, we utilized CEST and CPMG NMR experiments to investigate the thermal unfolding of the I59T variant under the same experimental conditions. This enabled us to not only analyze the otherwise invisible D state at lower temperatures and monitor the entire unfolding process accurately, but most significantly, to probe and identify a distinct intermediate (I) state. The I state is in exchange with both the N and D states, is only transiently and sparsely populated (up to ≈0.6%), and expands the previous pseudo‐two‐state model into a modified three‐state exchange model. The observed chemical shifts of the I state originate from the β‐domain, C‐helix and a residue from the N‐terminus, which are denatured at low temperatures and exhibit highly dynamic behavior (Figure S5c, Supporting Information).^[^ ^4^ ^]^ Such locally denatured regions are implicated in both intra‐ and inter‐molecular interactions, initiating the aggregation of the protein into amyloid fibrils.^[^ ^4^ ^]^

Our all‐atom unbiased MD simulations of the I state suggest that non‐native hydrogen bonds and amide‐π interactions account for the anomalously deshielded and shielded chemical shifts observed in the I state from CEST experiments. Specifically, the simulations indicate that the highly deshielded chemical shift of A42 arises from non‐native hydrogen bonding, while I56 and T59 show a strong preference for non‐native amide‐π hydrogen bonding with the aromatic rings of tryptophan and tyrosine. This is qualitatively consistent with their unusually shielded chemical shifts, which deviate significantly ‐ by ≈2.4 and ≈3.3 standard deviations, respectively ‐ below the averagechemical shifts for their residue types according to the BMRB statistics. Such deviations can be signatures of amide‐ring contacts in folded proteins,^[^ ^24^ ^]^ and have been previously observed in an SH3 folding intermediate.^[^ ^25^ ^]^ This could indicate that amide‐π hydrogen bonds are more prevalent than previously appreciated, potentially contributing to the stability of partially folded structures and folding intermediates. While classical all‐atom force fields do not preclude these interactions, their stability is likely underestimated.^[^ ^24^ ^]^ In agreement with this, we find clear evidence of such interactions in our simulations with both the C36m and a99sb‐disp force fields, although the magnitudes of the predicted ensemble‐averaged chemical shift changes are not entirely accurate. The limited ability of current all‐atom force fields to describe these interactions, combined with insufficient sampling, may account for discrepancies between computed and experimental chemical shifts.

Our all‐atom MD simulations do, however, recapitulate previously reported biophysical data on the I state, including the evidence of local unfolding in the β‐domain and C‐helix,^[^ ^3c^ ^]^ as well as the average folding pathway, characterized by early secondary structure formation in helices A and B and late folding of helices C and D and the β‐domain.^[^ ^22^ ^]^ The intermediate state ensemble retains stable A‐ and B‐helices but is more molten‐globule like in character due to reduced stability of tertiary interactions. While the β‐domain and C‐helix show the largest loss of native contacts, they remain relatively compact stabilized by non‐native contacts instead of undergoing complete local unfolding.

Recent atomic structures of human lysozyme amyloid fibrils have revealed polymorphism.^[^ ^26^ ^]^ Unlike the in vitro fibrils formed by the WT lysozyme under harsh conditions (85 °C, 300 rpm),^[^ ^26a^ ^]^ *ex vivo* fibrils from patients with the D87G mutation retain all four native disulfide bonds.^[^ ^26b^ ^]^ This suggests that the amyloid fibrils responsible for systemic amyloidosis are formed by partially unfolded protein intermediates with intact disulfide bonds, rather than by fully denatured proteins. Our previous and current work consistently support this, revealing the intermediate state with locally denatured β‐domain and C‐helix residues that mediate transient inter‐molecular interactions during the initial events of the amyloid fibril formation.^[^ ^4^ ^]^ Based on the flat structure of lysozyme monomers comprising the *ex vivo* amyloid fibrils,^[^ ^26b^ ^]^ we envisage that these intermediate state structures subsequently undergo local unfolding and rearranging of α‐domain residues, contributing to the formation of the remaining amyloid fibril architecture.

Our study suggests that the lysozyme intermediate is dynamic, sampling numerous non‐native interactions that likely prevent complete unfolding during aggregation. The observation of amide‐π hydrogen bonds, supported by the low chemical shifts of I56 and T59, highlights the role of non‐native interactions in stabilizing the I state and potentially contributing to the protein's amyloidogenicity. We propose that intra‐chain amide‐π hydrogen bonds (spanning more than three amino acids) are more prevalent in partially folded states than in well‐ordered protein structures. In folded proteins, hydrogen bonding is maximized to be energetically more favorable, and aromatic sidechains are tightly packed with hydrophobic residues in the core. In partially folded states, reduced secondary structure exposes more amides and aromatic rings, facilitating the formation of amide‐π hydrogen bonds.

To prevent human lysozyme aggregation, molecules that stabilize the native state, such as ligands^[^ ^26b^ ^]^ or nanobodies,^[^ ^27^ ^]^ have been explored. However, given the critical role of the intermediate state in initiating aggregation into amyloid fibrils, compounds that specifically destabilize this state could also serve as effective inhibitors of amyloidosis. Aromatic compounds, for instance, might compete with residues in the protein core for amide‐π interactions, thereby destabilizing the I state. Our structural insights may provide a valuable foundation for designing targeted bindinger molecules to inhibit human lysozyme amyloid formation.

## 4 Experimental Section

All chemicals and reagents were purchased from Sigma Aldrich Ltd. (Gillingham, UK) unless otherwise stated.

### Protein Expression and Purification

Lysozyme variants were expressed in *Pichia pastoris* and purified as described previously.^[^ ^28^ ^]^ Protein purity (>95%) was confirmed using SDS‐PAGE, and molecular masses were confirmed by mass spectrometry.

### Circular Dichroism Spectroscopy

CD spectroscopy experiments were performed using a Jasco J‐810 spectropolarimeter (JASCO Ltd, Great Dunmow, UK) equipped with a Peltier temperature controller. Protein samples (20 µm) were dissolved in phosphate buffer (50 mm, pH 1.2) and analyzed in a 0.1 cm pathlength cuvette. Thermal denaturation was monitored at 222 or 270 nm with temperature increments from 5 to 95 °C (1 °C min^−1^). Ellipticity values were normalized to the fraction of denatured state protein and fitted to a two‐state unfolding model assuming linear baselines for both native and denatured states as described previously.^[^ ^4^ ^]^

### Thermal Unfolding Monitored by 2D NMR

I59T human lysozyme (200 µm) was prepared in phosphate buffer (50 mm, pH 1.2) with 90% H_2_O/10% D_2_O. 2D ^1^H‐^15^N HSQC spectra were recorded at temperature intervals of 2.5 or 5 °C between 5 and 60 °C on a Bruker Avance 700 MHz NMR spectrometer (University of Cambridge) and processed with NMRPipe^[^ ^29^ ^]^ and Sparky.^[^ ^30^ ^]^ Cross peak intensities were calculated by measuring the peak volumes using FuDA (http://www.biochem.ucl.ac.uk/hansen/fuda). The populations of the native (N) and denatured (D) states for each residue were determined by referencing the intensities of the N (at 7.5 °C) and D (57.5 °C) state, respectively.^[^ ^3c^ ^]^ Peak assignments in the ^1^H‐^15^N HSQC spectra were performed using previously reported assignments.^[^ ^4^ ^]^

### Peak Assignments by 3D NMR

Isotopically double‐labeled (^13^C, ^15^N) I59T was generated as described previously.^[^ ^4^ ^]^ Backbone and side‐chain resonances for the N and D states were acquired and assigned at 25 and 45 °C, respectively. For N state resonance assignments, CCCONH (2048 × 64 × 128 complex points and SW of 14 (^1^H), 35 (^15^N), 75 (^13^C) ppm),^[^ ^31^ ^]^ HCCH‐TOCSY (2048 × 64 × 128, 14 (^1^H), 12 (^1^H), 75 (^13^C) ppm),^[^ ^32^ ^]^ HCCH‐COSY (2048 × 128 × 64, 14 (^1^H), 12 (^1^H), 75 (^13^C) ppm),^[^ ^32^ ^]^ HNCO (2048 × 64 × 64, 14 (^1^H), 30 (^15^N), 12 (^13^C) ppm),^[^ ^33^ ^]^ HN(CO)CACB (2048 × 64 × 128, 14 (^1^H), 30 (^15^N), 62 (^13^C) ppm)^[^ ^33^ ^]^ experiments were performed on a Bruker Avance 700 MHz spectrometer (University of Cambridge). For D state resonance assignments, HNCO (2048 × 80 × 96, 15 (^1^H), 22 (^15^N), 10 (^13^C) ppm),^[^ ^33^ ^]^ was recorded on a 700 MHz Bruker Avance III HD (Francis Crick Institute) and H(CC)CONH (3072 × 64 × 64, 18 (^1^H), 21 (^15^N), 10 (^1^H) ppm),^[^ ^31^ ^]^ (H)CCCONH (3072 × 64 × 64, 18 (^1^H), 21 (^15^N), 10 (^1^H) ppm)^[^ ^31^ ^]^ were recorded on a 800 MHz Bruker Avance III spectrometer (University College London) equipped with a TXI cryoprobe.

### CEST NMR

^15^N CEST experiments were performed with two ^15^N B1 fields (during T_EX_) of 15 and 60 Hz at 11.7, 16.4, 18.8 and 22.3 T. The weak B1 fields were calibrated using a sensitivity‐enhanced ^15^N‐*R*_1_ measurement.^[^ ^34^ ^]^ CEST experiments were performed at 25–45 °C. At 11.7 T, 42 2D ^1^H‐^15^N HSQC spectra were acquired with a series of ^15^N offsets ranging between 100.78 and 132.23 ppm obtained in increments of 0.79 ppm (40 Hz). Each 2D data set comprised 2048 × 128 complex points in the ^1^H and ^15^N dimensions and was recorded with 8 scans, an inter‐scan delay of 1.1 s, a saturation time (T_EX_) of 0.3 s. A reference spectrum was acquired with T_EX_ = 0 s. At 16.4 T, 82 2D ^1^H‐^15^N HSQC spectra were acquired with ^15^N offsets ranging between 102 and 132 ppm obtained in increments of 0.38 ppm (27 Hz). Each 2D data set comprised 2048 × 128 complex points in the ^1^H and ^15^N dimensions and was recorded with 4 scans, an inter‐scan delay of 1 s, T_EX_ of 0.4 s. At 18.8 T, 62 2D ^1^H‐^15^N HSQC spectra were acquired with ^15^N offsets ranging between 100.76 and 132.36 ppm obtained in increments of 0.49 ppm (40 Hz). Each 2D data set comprised 3072 × 128 complex points in the ^1^H and ^15^N dimensions and was recorded with 4 scans, an inter‐scan delay of 1.3 s, T_EX_ of 0.35 s. At 22.3 T, 82 2D ^1^H‐^15^N HSQC spectra were acquired with ^15^N offsets ranging between 102 and 132 ppm obtained in increments of 0.38 ppm (37 Hz). Each 2D data set comprised 2048 × 128 complex points in the ^1^H and ^15^N dimensions and was recorded with 8 scans, an inter‐scan delay of 0.95 s, T_EX_ of 0.4 s.

^13^C CEST experiments for methyl carbons were performed at 35 °C, 22.3 T, using a 13C B1 field strength of 25 Hz with T_EX_ = 0.3 s. 81 2D ^1^H‐^13^C HSQC spectra were acquired with ^13^C offsets ranging between 12.23 and 31.05 ppm obtained in increments of 0.21 ppm (50 Hz). Each 2D data set comprised 4096 × 220 complex points in the ^1^H and ^13^C dimensions and was recorded with 4 scans, an inter‐scan delay of 1.6 s, T_EX_ of 0.3 s. 13Cα CEST experiments were performed at 35 °C, 18.8 T, using a ^13^C B1 field strength of 30 Hz with TEX = 0.3 s. 77 2D ^1^H‐^13^C HSQC spectra were acquired with ^13^C offsets ranging between 50.43 and 69.06 ppm obtained in increments of 0.25 ppm (50 Hz). Each 2D data set comprised 3072 × 256 complex points in the ^1^H and ^13^C dimensions and was recorded with 4 scans, an inter‐scan delay of 1 s, T_EX_ of 0.3 s.

^1^H CEST experiments for methyl sidechains were performed at 35 °C, 22.3 T, using a Z‐Z exchange‐based pulse sequence with a ^1^H B1 field strength of 60 Hz with T_EX_ = 0.3 s. 145 2D ^1^H‐^13^C HSQC spectra with ^1^H offsets ranging between −2.84 and 4.73 ppm obtained in increments of 0.05 ppm (50 Hz). Each 2D data set comprised 4096 × 200 complex points in the ^1^H and ^13^C dimensions and was recorded with 4 scans, an inter‐scan delay of 1s. ^1^Hα CEST experiments were performed at 35 °C, 22.3 T with a ^1^H B1 field strength of 45 Hz with T_EX_ = 0.3 s. 82 2D ^1^H‐^13^C HSQC spectra with ^1^H offsets ranging between 2.96 and 5.94 ppm obtained in increments of 0.04 ppm (35 Hz). Each 2D data set comprised 4096 × 96 complex points in the ^1^H and ^13^C dimensions and was recorded with 4 scans, an inter‐scan delay of 1 s.

### CPMG Relaxation Dispersion NMR

CPMG relaxation dispersion profiles were recorded at 16.4, 18.8 and 21.1 T at 37.5 and 45 °C as described previously.^[^ ^35^ ^]^ A constant‐time CPMG interval (40 ms) with ^1^H continuous‐wave decoupling along with 20 ν_CPMG_ values ranging from 25 to 1000 Hz were used. Each 2D data set comprised 2048 × 128 complex points in the ^1^H and ^15^N dimensions and was recorded with 8 scans, an inter‐scan delay of 1 s.

### Rotating Frame (R_1ρ_) Relaxation Dispersion NMR

On‐ and off‐resonance *R*_1ρ_ measurements were performed at 37.5 °C for the denatured peak of G129 at a field strength of 16.4 T as previously described.^[^ ^36^ ^] 15^N RF field strengths were calibrated by pulse nutation experiments. On‐resonance *R*_1ρ_ experiments were performed at spin‐lock field strengths (ω_1_/2π) ranging from 100 to 2000 Hz. Off‐resonance experiments were recorded at three spin‐lock field strengths (200, 1000, and 2000 Hz) with 12 to 20 offsets. Each data set comprised 2048 complex points in the 1H dimension and was recorded with 64 scans, an inter‐scan delay of 1 s.

### NMR Data Fitting

^15^N CEST and CPMG data were processed using FuDA and fitted using the ChemEx software package (http://www.github.com/gbouvignies/chemex). For two‐state model, CEST data from 109 residues in both the N and D states were globally fitted to a two‐state unfolding model (N‐D) at 25–37.5 °C (Figure 1E). For three‐state model, both the N and D state CEST and CPMG data from 15 residues (total dataset: 525; Figure S2, Supporting Information) were globally fitted to a triangular model as well as three sequential models (N‐I‐D, N‐D‐I, I‐N‐D). These results were compared with those from a global fitting excluding the 60 Hz B1 field data from F57, D67, L84 and A92 (reduced dataset: 468). Excluding the 60 Hz B1 field data improved the fitting, as indicated by a lower reduced χ^2^, but the resulting kinetic parameters remained effectively unchanged.

On‐ and off‐resonance *R*_1ρ_ data were globally fit to the Trott and Palmer equation:^[^ ^37^ ^]^

(1) $$R_{1\rho }=R_{1}cos^{2}\theta +\left(R_{2}+R_{ex}\right)sin^{2}\theta$$

where

(2) $$R_{ex}=\frac{p_{B}\Delta \omega^{2}k_{ex}}{{\left(\delta_{A}+\Delta \omega \right)}^{2}+{\omega_{1}}^{2}+{k_{ex}}^{2}}$$

with *ω*_1_ is the ^15^N spin‐lock field strength, *δ*_A_ is the ^15^N resonance offset of the A state and ∆*ω* is the frequency difference between state A and B in the asymmetric population limit (*p*_A_≫*p*_B_).

### Ratchet‐and‐Pawl MD Simulations

All simulations in this study were run using GROMACS (v2018 and v2021).^[^ ^38^ ^]^ Starting structures for rMD folding simulations were generated using short, all‐atom structure‐based model^[^ ^39^ ^]^ simulations where all native contacts were deleted, resulting in rapid unfolding and the generation of random sterically allowed conformers. All four disulfide bridges were kept oxidized. Resulting structures were centered in a dodecahedron box with a padding of 1.0 nm. Following solvation and neutralization using chlorine ions, structures were energy minimized using the steepest‐descent algorithm and equilibrated for 500 ps first in the NVT (308 K) and then NPT (308 K, 1 bar) ensemble using heavy atom position restraints with a force constant of 1000 kJ mol^−1^ nm^−1^. Subsequently, the structures were relaxed without positionrestraints in the NPT ensemble (308 K, 1 bar) for 1 ns. The LINCS algorithm^[^ ^40^ ^]^ was used to constrain all bonds to hydrogens together with a timestep of 2fs and leap‐frog algorithm for integration. The temperature was controlled using the velocity rescaling algorithm^[^ ^41^ ^]^ with a time constant of 0.1 ps. During equilibration, the pressure was maintained using the Berendsen barostat^[^ ^42^ ^]^ with a compressibility of 4.5 × 10^−5^ bar^−1^ and the Parrinello‐Rahman algorithm during the 1 ns relaxation and following production rMD simulations.^[^ ^43^ ^]^ Simulations were parameterized using the CHARMM36m (C36m)^[^ ^20^ ^]^ and a99sb‐disp^[^ ^21^ ^]^ force fields. To mimic the low pH conditions of the NMR experiments, all titratable groups were protonated accordingly. Nonbonded van der Waals interactions were treated to a cut‐off at 1.2 nm (with a switching function from 1.0 nm for C36m). Electrostatics were calculated to a cut‐off of 1.2 nm and long‐range electrostatics were computed using the Particle Mesh Ewald (PME) method.^[^ ^44^ ^]^

Production simulations employing the rMD algorithm were run for 5 ns with a 2fs timestep at 350 K and 1 bar in the NPT ensemble. In rMD simulations a history‐dependent biasing force is introduced when the protein attempts to backtrack along its folding trajectory defined as^[^ ^13^, ^19^ ^]^

(3) $$F_{rMD}=\left\{\begin{array}{c} \frac{\kappa }{2}{\left(\rho \left(t\right)-\;\rho_{m}\left(t\right)\right)}^{2},\;\rho \left(t\right)>\;\rho_{m}\left(t\right)\\ 0,\;\rho \left(t\right)\;\leq \;\rho_{m}\left(t\right) \end{array}\right\}$$

where ρ(*t*) =  (*CV*(*t*) − *CV_target_*)^2^ and $\rho_{m}\left(t\right)=\min_{0\to t}\left(CV\right)$. *κ* is the force constant controlling the strength of the biasing force applied, *CV(t)* is the value of a chosen collective variable at time t and *CV_target_* is the defined target value of the collective variable (in this case the native state). This work used a κ value of 1 × 10^−9^ kJ mol^−1^. The collective variable (CV) for ratcheting used here is the difference in the contact map of the structure at time t, *C_ij_* (X), and the native state, *C_ij_* (*X_native_*), defined as^[^ ^19a,b^ ^]^

(4) $$CV=\sum_{\left|i-j\right|>35}{\left(C_{ij}\left(X\right)-\;C_{ij}\left(X_{native}\right)\right)}^{2}$$

(5) $$C_{ij}\left(X\right)=\frac{1-\;{\left(\frac{r_{ij}}{r_{0}}\right)}^{6}}{1-\;{\left(\frac{r_{ij}}{r_{0}}\right)}^{10}}$$

All heavy atom indices i and j are included in the contact map and *r_ij_* is the distance between heavy atoms while *r*_0_ is 0.75 nm. All distances up to and including 1.2 nm in the native crystal structure (PDB:2MEH^[^ ^15^ ^]^) are included. This work simulated a total of 36 folding trajectories (each 5 ns in length) with different random initial velocities for 50 different input structures with each force field, yielding a total of 9 µs of rMD data per force field.

To calculate the kinetic folding free energy landscape, this work extracted an ensemble of folding trajectories that reached the native state (defined as Cα‐RMSD ≤ 3.0 Å relative to the crystal structure). For these ensembles of folding trajectories, this work estimated the free energy landscape as a function of Cα‐RMSD and Q (the fraction of native contacts, defined as in^[^ ^45^ ^]^ with λ = 1.5), smoothed by a Gaussian filter with σ = 2. The intermediate basin was defined as a local minimum with structures below 11 kJ mol^−1^. This work then clustered all structures from the intermediate basin using the GROMOS algorithm,^[^ ^46^ ^]^ the Cα‐RMSD as a similarity measure and 5 Å as cut‐off value using.

### Unbiased MD Simulations of the Intermediate State

This work chose two starting structures per force field from the intermediate clusters where the C‐helix is unfolded and lacks tertiary contacts with the α‐domain. Each starting structure was centered in a dodecahedron box with 1.2 nm of padding and solvated/neutralized as before for rMD simulations. This work parameterized the simulations with the respective force fields and protonated all titratable groups. Simulation boxes were energy minimized by the steepest descent algorithm and then equilibrated for 500 ps in the NVT ensemble (308 K) followed by 500 ps in the NPT ensemble (308 K, 1 bar). This work employed the same simulation algorithms and cut‐off values as for the rMD simulations. To facilitate sampling of longer timescales, this work ran simulations using hydrogen mass repartitioning (HMR),^[^ ^47^ ^]^ allowing a timestep of 4 fs. Four production simulations of 10 µs were launched for each starting structure with different initial velocities. This yielded a total of 160 µs of MD for the intermediate state. Control simulations for each force field of the native state were initiated from the crystal structure^[^ ^15^ ^]^ and run equivalently for 4 × 2.5 µs with different initial velocities.

### Ensemble Analysis

This work used the python packages MDAnalysis^[^ ^48^ ^]^ and MDTraj^[^ ^49^ ^]^ for analysis of the ensembles involving atomic coordinates. The fraction of native contacts was calculated as defined by Best et al.^[^ ^45^ ^]^

(6) $$Q\left(X\right)=\frac{1}{N}\sum_{ij}\frac{1}{1+e^{\left(\beta \left(r_{i,j}-\lambda {r^{0}}_{i,j}\right)\right)}}$$

where *r_i,j_* and *r^0^_i,j_* are the distances between atoms *i* and *j* in frame X and the template structure, respectively, β modulates the smoothness of the switching function (default value 5 Å^−1^used) and λ is a factor allowing for fluctuations of the contact distance (set to 1.5).

Contact maps were calculated using distances between all heavy atoms and a cut‐off distance of 5.0 Å. Secondary structure populations were computed with the DSSP algorithm^[^ ^50^ ^]^ with the implementation in MDTraj. GROMACS^[^ ^38^ ^]^ was used to calculate the solvent‐accessible surface area (SASA) using all protein atoms. Backbone chemical shifts were calculated from MD ensembles using SHIFTX2, which has the highest reported accuracy.^[^ ^23^ ^]^ To compare the global agreement with chemical shifts from different nuclei, this work calculated a chemical shift score defined as the summed RMSD for each nuclei normalized by the average error of the method for the respective nucleus. Finally, amide‐π hydrogen bonds were defined by the following criteria:

1. An amide proton to ring center of mass (COM) distance of less than 4.5 Å
2. The angle between the ring normal vector and vector formed by the amide proton and ring COM must be less than 54.7 degrees (i.e., the amide is perpendicular to the ring plane)
3. The angle formed by the amide nitrogen, proton and ring COM must be higher than 120.0 degrees (hydrogen bonding directionality)
4. The amide proton is not involved in another non‐aromatic hydrogen bond (normal hydrogen bonds are defined by amide proton ‐ acceptor distances of less than 2.5 Å and angles higher than 120.0 degrees).

### MD Simulations for Folding Process

To validate the average folding pathway observed in the rMD simulations, this work compared the folding simulations to biophysical data available in the literature. First, this work assessed the order of secondary structure formation, focusing on the main helices A, B, C, D, and the β‐sheet (Figure S8C,D, Supporting Information). Pulse deuterium labelling studies revealed that during non‐oxidative refolding of HuL helices A and B are protected rapidly from exchange, before the other secondary structure elements.^[^ ^22^ ^]^ This is followed by formation of secondary structure in helices C and D, and afterward in the β‐sheet.^[^ ^22^ ^]^ Consistent with these experiments, this work observes for both force fields that the β‐sheet forms the lowest amount of secondary structure on average in the folding simulations, and it forms more slowly than the main helices (Figure S8D, Supporting Information). Furthermore, helix D is predicted to form less structure and more slowly than helices A and B, also consistent with experiments. However, helices A‐C appear to form similar amounts of structure and follow similar kinetics on average, suggesting in simulations helix C is too stable and forms too early. This work also assessed the order of tertiary contact formation of the main helices and β‐sheet, which showed that for both force fields the simulations predict rapid collapse of formation of tertiary native contacts for all structural elements, except helix C which is delayed (Figure S8E, Supporting Information). This observation is consistent with previous NMR data showing that the intermediate observed during thermal unfolding mutant has an unfolded C‐helix and β‐sheet.^[^ ^3c^ ^]^

### MD Simulations for Trp Residues SASA

To assess the accuracy of tertiary structure formation in the simulations further, this work computed the solvent accessibility of two tryptophan indole groups (Trp109 and Trp112), which were seen by pulse labelling NMR to follow different protection kinetics. Consistent with experiments,^[^ ^22^ ^]^ the simulations predict more rapid burial of Trp112 than Trp109 (Figure S8F,G, Supporting Information). This is also consistent with late folding of the C‐helix against the rest of the protein, since Trp112 reports mainly on contacts between helices D and B and Trp109 on contacts with the C‐helix (Figure S8F, Supporting Information). Last, stopped‐flow CD experiments showed that during refolding ≈50% of native like signal in the near UV range develops rapidly, followed by slower development of the remaining native signal.^[^ ^22^ ^]^ This suggests that two out of the four buried tryptophan residues are buried prior to burial of the other two. Indeed, this behavior is also predicted by the simulations with both force fields, showing that Trp28 and Trp109 are buried more slowly than Trp64 and Trp112 (Figure S8H, Supporting Information). This is consistent with Trp28 and Trp109 reporting on tertiary contacts with the C‐helix and partial exposure of these sidechains in the intermediate state.

### Secondary Structure Stability

Next, this work assessed the stability of secondary structure in the unbiased MD simulations and found that helices A and B maintained the most helical structure, while helix C was more variable and can exhibit large amounts of helicity but also almost complete unfolding. Helix D also has a reduced helicity (Figure 4D; Figure S9C, Supporting Information). This is broadly consistent with the C‐helix forming at least some secondary structure before the β‐domain during refolding,^[^ ^22^ ^]^ and thus before tertiary contacts are established, but also partial unfolding of helix C in the intermediate state. The β‐domain, in contrast to the native state, shows a significant reduction in the amount of β‐sheet structure, consistent with unfolding of this domain (Figure 4D; Figure S9C, Supporting Information).

### Statistical Analysis

The standard deviation (SD) from the global fitting of the CEST and CPMG RD data was used as the uncertainty for the kinetic parameters and chemical shifts of the three states. χ^2^, BIC, and AIC values for the global fitting were calculated using ChemEx software package. All MD analyses of the folding pathways (rMD) and the I state ensemble (unbiased MD) report the mean across all trajectories; no further quantities were calculated due to the limited sampling. No data were excluded.

## Conflict of Interest

The authors declare no conflict of interest.

## Author Contributions

M.A., J.O.S., and C.A.W. contributed equally to this work. M.A., J.O.S., C.A.W., T.W., J.C., and J.R.K. conceived and designed the project. M.A., C.A.W., and A.M.F. performed the NMR experiments. M.A. and C.A.W. analyzed the NMR data with input from A.M.F., J.C., and J.R.K. J.O.S. and T.W. carried out MD simulations and analyzed the data with input from M.A. and J.C. M.A. performed CD thermal unfolding experiments and analyzed the data. M.A., J.O.S., C.A.W., T.W., and J.R.K. prepared the manuscript with input from all other authors. J.C. and J.R.K. sourced the funding. M.A., J.C., and J.R.K. supervised the project.

## Supporting information



Supporting Information

Supplemental Movie 1

Supplemental Movie 2

Supplemental Movie 3

Supplemental Movie 4

Supplemental Movie 5

Supplemental Movie 6

## Data Availability

The data that support the findings of this study are available from the corresponding author upon reasonable request.
