# Supplementary material for: Amyloid Forming Human Lysozyme Intermediates are Stabilized by Non‐Native Amide‐π Interactions
Source: Adv Sci (Weinh). 2025 Jun 25;12(34):e03957. doi: 10.1002/advs.202503957 (PMC12442690; doi:10.1002/advs.202503957)
Supplement: Supplementary file 1 — Supporting Information [file ADVS-12-e03957-s003.docx]

Supporting Information

Amyloid Forming Human Lysozyme Intermediates are Stabilized by Non-Native Amide-π Interactions

Minkoo Ahn*, Julian O. Streit, Christopher A. Waudby, Tomasz Włodarski, Angelo Miguel Figueiredo, John Christodoulou*, Janet R. Kumita*

**Table S1**. C_α_ and C_β_ chemical shifts of cysteine residues in the native and denatured states at 35 ºC. All native cysteine residues are oxidized as expected. Cβ chemical shifts of C6, C65 and C95 confirm that three disulfide bonds (C6-C128, C65-C81, C77-C95) are intact (oxidized).^[1]^ The chemical shifts for C30 and C116 could not be assigned (n/a) due to overlapping peaks in the denatured state spectra. However, their disulfide bond is predicted to be intact, as the C77-C95 bond (the weakest of the four^[2]^) remains intact.

| residue | Cβ | | Cα | |
| --- | --- | --- | --- | --- |
|  | native | denatured | native | denatured |
| 6 | 32.861 | 40.378 | 54.19 | 56.388 |
| 30 | 44.873 | n/a | 60.646 | n/a |
| 65 | 45.481 | 41.272 | 53.087 | 56.37 |
| 77 | 39.277 | n/a | 55.933 | n/a |
| 81 | 37.715 | n/a | 56.846 | 56.93 |
| 95 | 34.979 | 41.924 | 55.3 | 56.551 |
| 116 | 44.066 | n/a | 54.664 | n/a |
| 128 | 34.931 | n/a | 51.92 | 55.632 |

**Table S2**. ^15^N Chemical shifts for the native, intermediate and denatured states from three-state global fitting of CEST and CPMG RD data.

| residue | native | intermediate | denatured |
| --- | --- | --- | --- |
| V2 | 127.126 | 125.530 | 122.660 |
| A42 | 123.179 | 131.618 | 124.343 |
| T43 | 116.778 | 114.898 | 112.503 |
| S51 | 112.427 | 114.661 | 115.928 |
| T52 | 116.708 | 119.195 | 114.795 |
| D53 | 123.668 | 122.79 | 120.419 |
| G55 | 111.119 | 112.842 | 109.229 |
| I56 | 120.229 | 110.276 | 119.041 |
| F57 | 114.345 | 109.296 | 121.638 |
| Q58 | 114.686 | 117.525 | 120.578 |
| T59 | 117.043 | 108.762 | 114.082 |
| N60 | 126.534 | 113.113 | 120.084 |
| D67 | 130.773 | 124.713 | 119.905 |
| L84 | 116.286 | 120.271 | 118.587 |
| A92 | 124.211 | 127.199 | 123.656 |

**SI Movies** **1-6**.

S1. N state a99sb-disp

S2. N state C36m

S3. I state a99sb-disp (1 input structure, 4 x 10 μs)

S4. I state a99sb-disp (1 input structure, 4 x 10 μs)

S5. I state C36m (1 input structure, 4 x 10 μs)

S6. I state C36m (1 input structure, 4 x 10 μs)


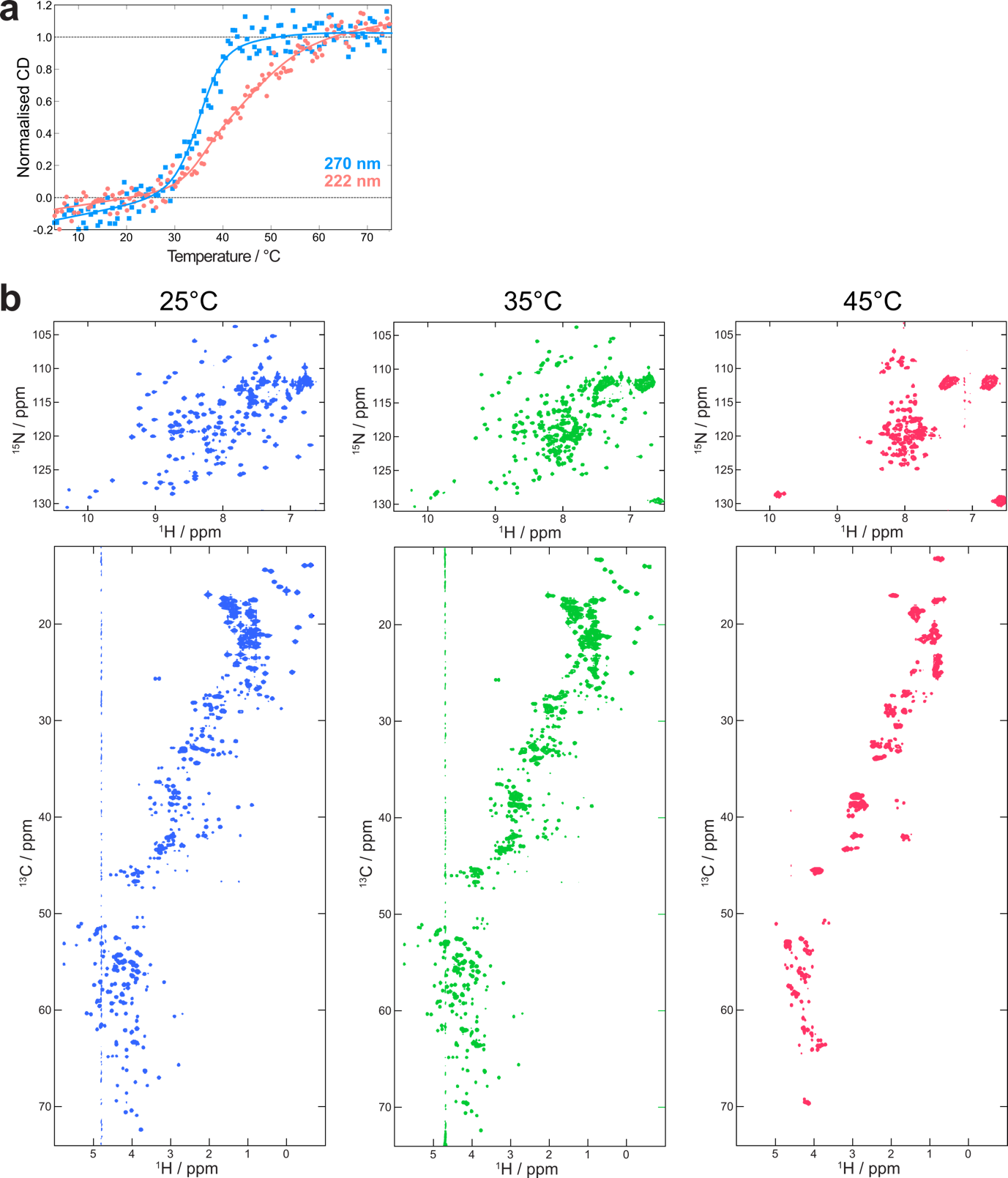


**Figure S1.** Thermal unfolding of I59T monitored by 2D NMR and CD spectroscopy. (a) Normalized signal from far-UV (222 nm) and near-UV (270 nm) CD thermal unfolding of I59T recorded at pH 1.2. Solid lines represent fitted curves. (b) 2D ^1^H-^15^N and ^1^H-^13^C spectra of I59T at pH 1.2, recorded at 25, 35 and 45 ºC on an 800 MHz spectrometer.


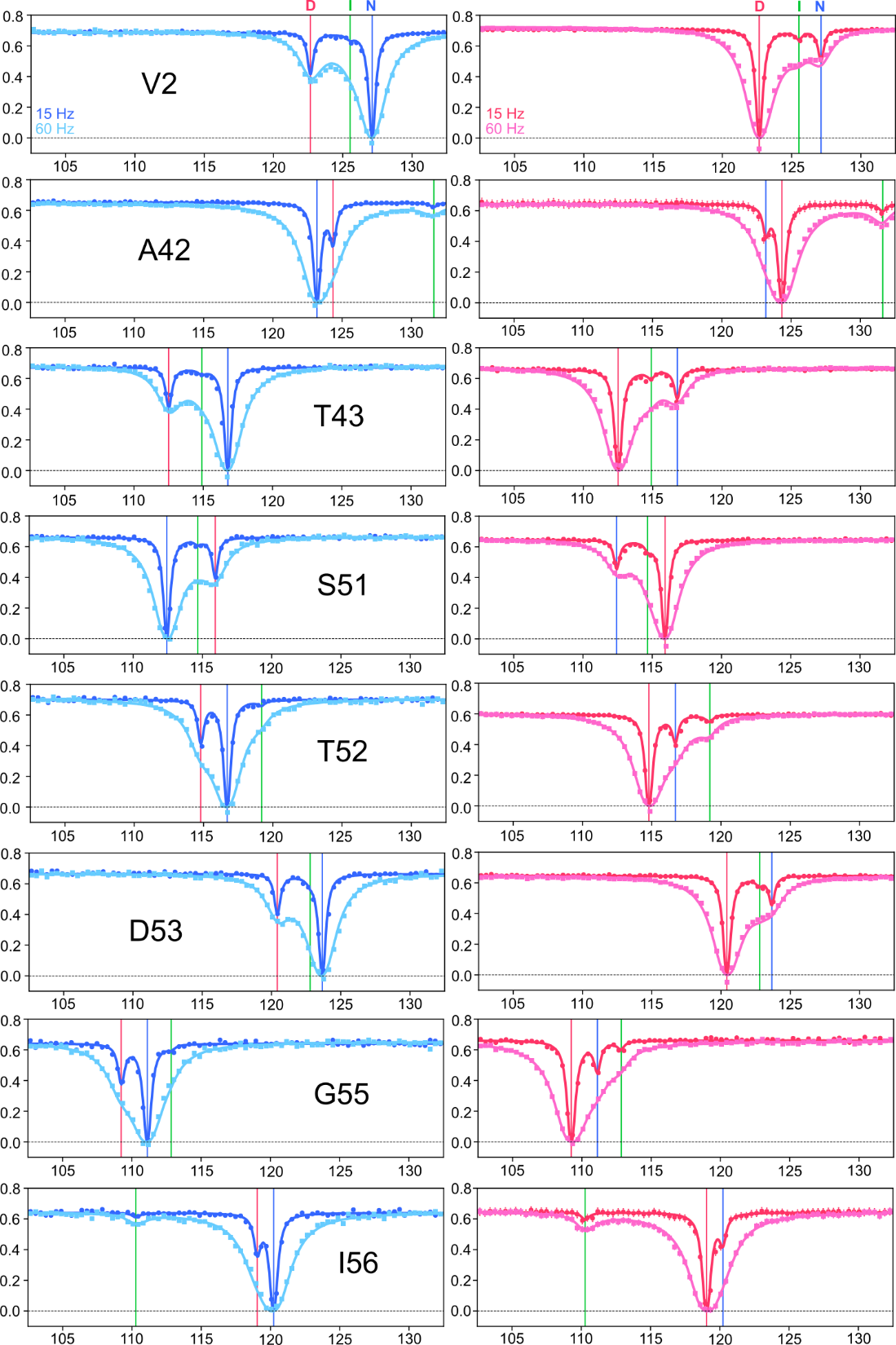


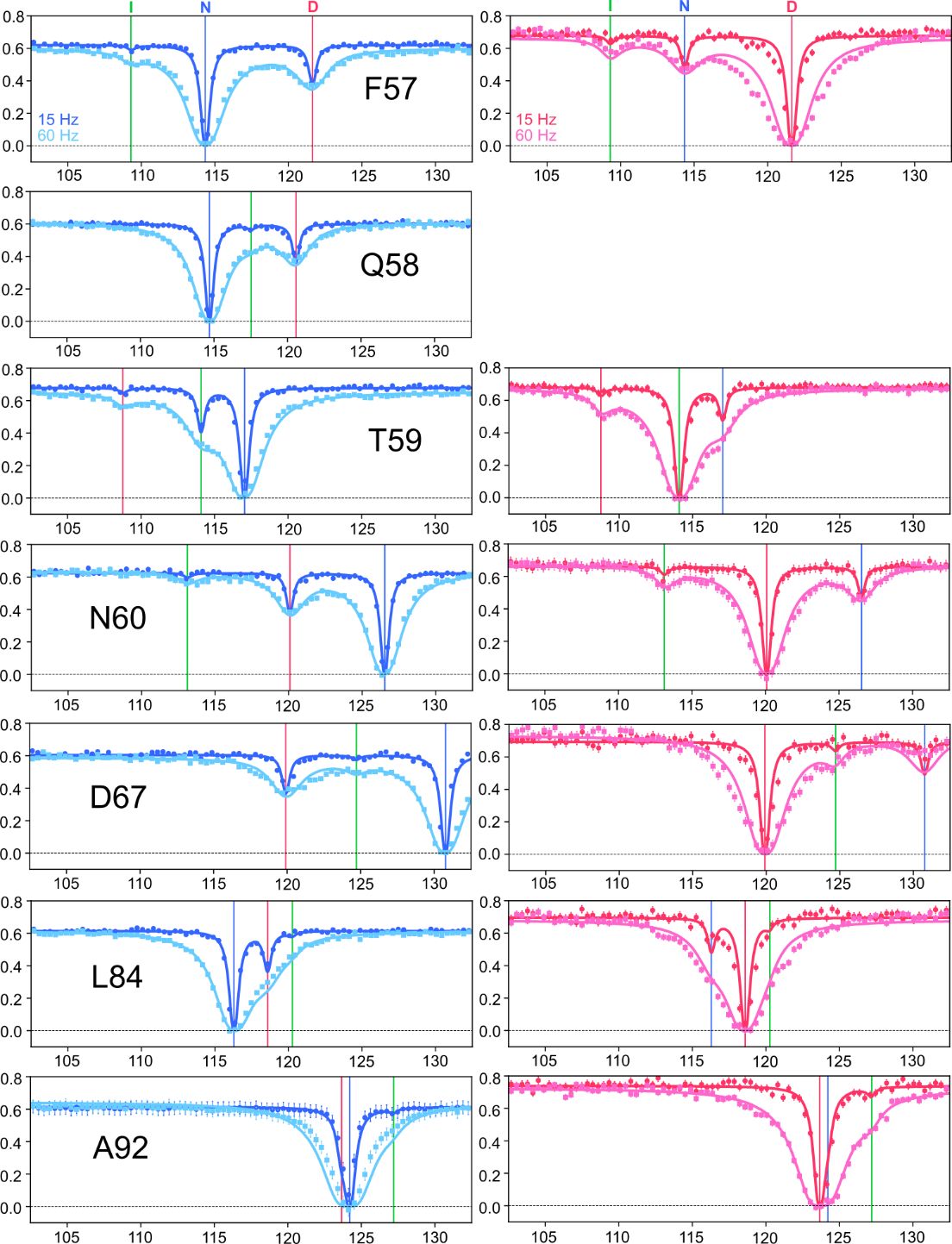


**Figure S2.** ^15^N CEST profiles of the native and denatured peaks for 15 residues that reveal the intermediate state, recorded at 800 MHz and 35 ºC using 15 Hz and 60 Hz B1 fields. The data were analyzed using three-state global fitting, including all other data sets collected at various temperatures (25, 30, 35, 37.5, 40, and 45 ºC), field strengths (500, 700, 800, and 950 MHz) and CPMG RD data (700, 800, 900, and 950 MHz), as shown in Figure S3. Blue, red and green vertical lines indicate the chemical shifts of the native, denatured and intermediate states, respectively. The data for the denatured peak of Q58 were not included in the global fitting due to significant overlap with other peaks.


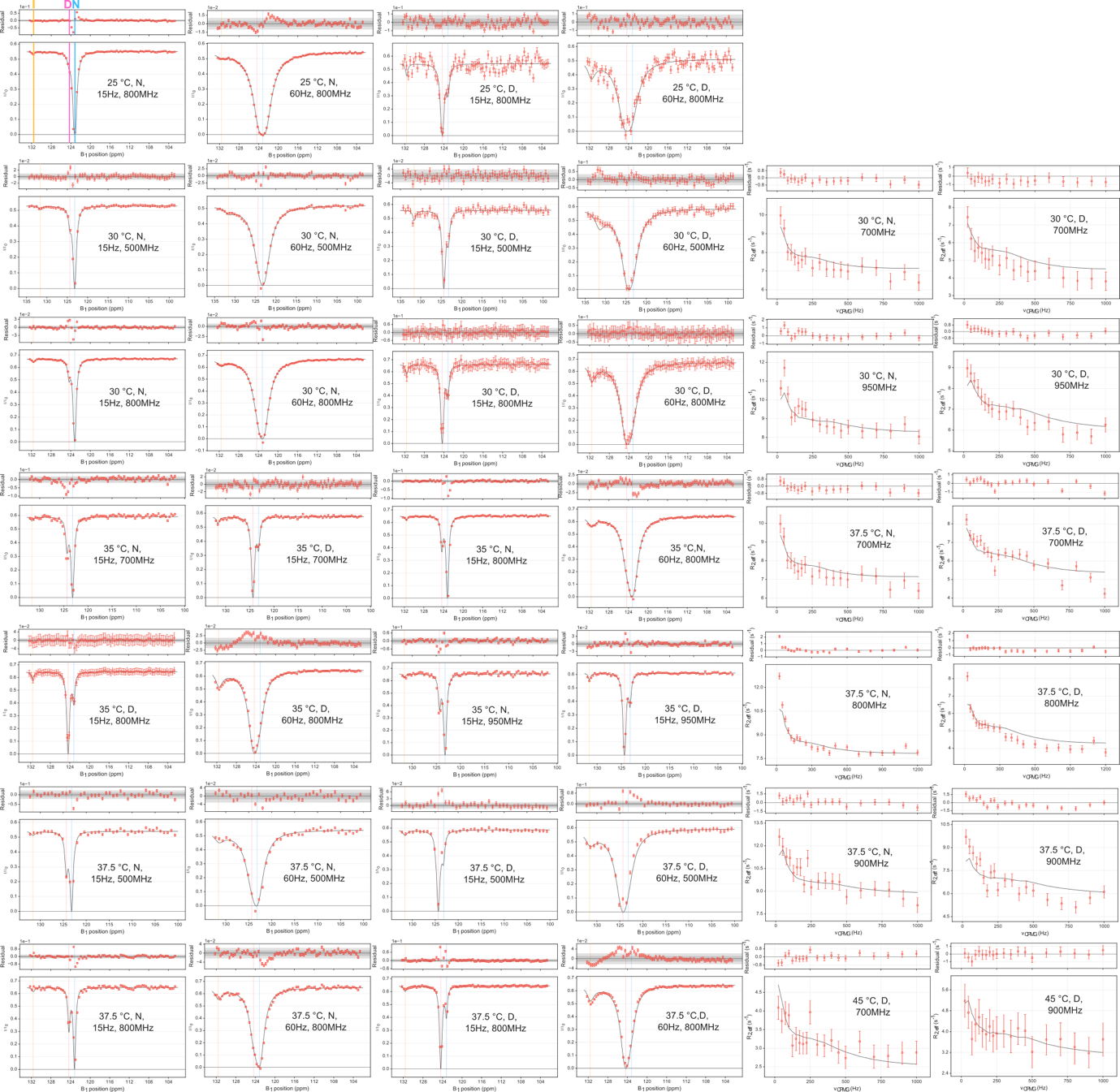


**Figure S3.** Global fitting of ^15^N CEST and CPMG RD data to a three-state unfolding model. Data for residue A42 collected at various temperatures and field strengths used for ChemEx data fitting are shown as an example. N and D represent the data from the native and denatured peaks. Blue, red and yellow solid vertical lines in CEST profiles represent the chemical shifts of the native, denatured and intermediate states, respectively.


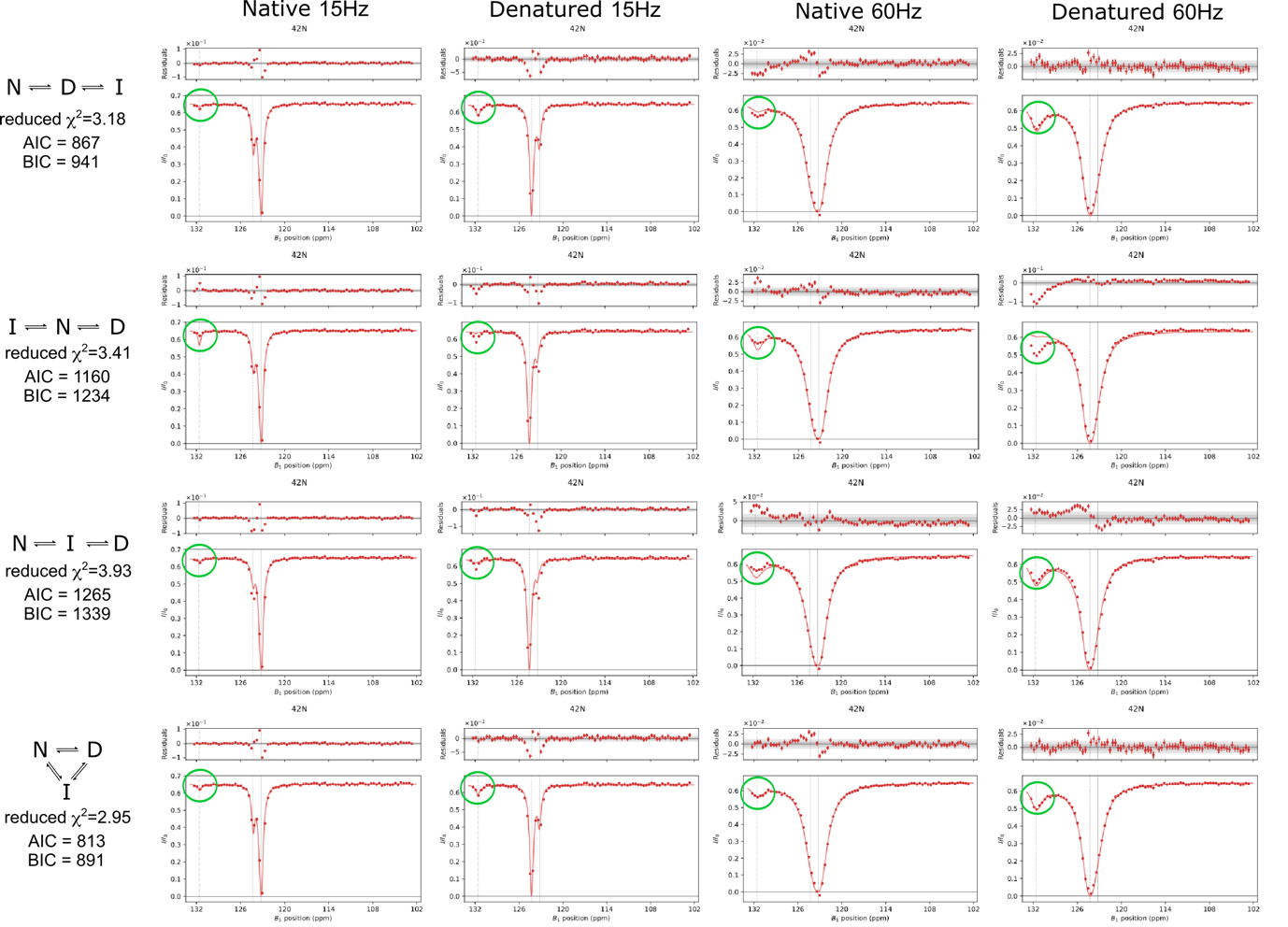


**Figure S4.** Global fitting of ^15^N CEST and CPMG data of A42 to four three-state exchange models. The native and denatured peak CEST profiles recorded with 15 and 60 Hz B1 field at 800 MHz and 35 ºC are shown for comparison. Green circles indicate the CEST dips corresponding to the intermediate (I) state. The triangular model (bottom) shows the best fit, as evidenced by the lowest χ^2^, AIC and BIC values.


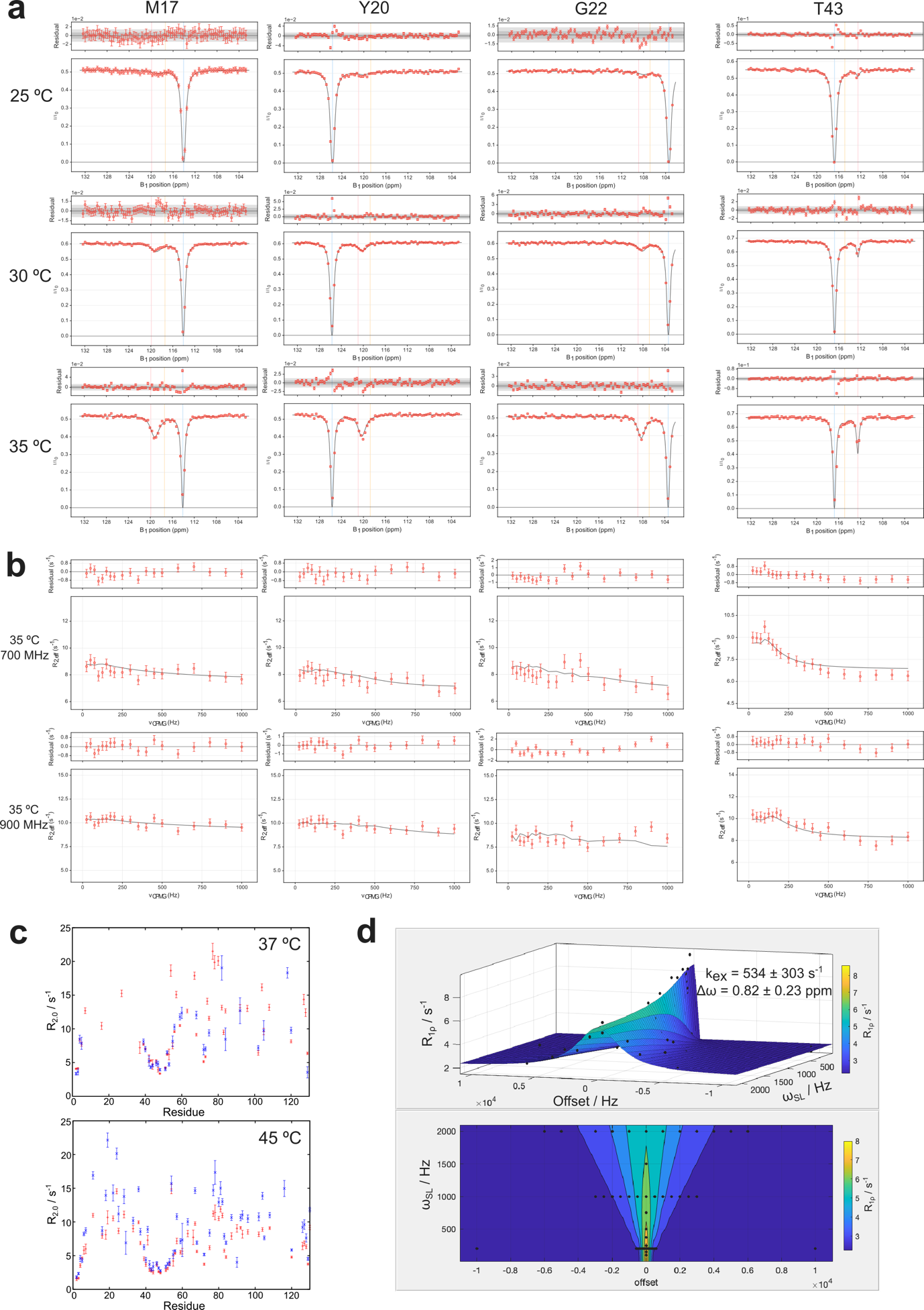


**Figure S5.** Probing the dynamics of α-domain residues. CEST (a) and CPMG (b) data the N state peaks of α-domain residues (helix A) fitted to a three-state unfolding model. Three residues (M17, Y20 and G22) from helix A show distinct behaviour compared to the β-domain residue (T43). CEST data are recorded at 800 MHz. (c) *R*_2,0_ values from the fitting of CPMG data for D state peaks at 37 (top) and 45 (bottom) ºC, recorded at 700 (red) and 900 (blue) MHz. (d) On- and off-resonance *R*_1ρ_ relaxation dispersion data for the denatured peak of G129 were fit to the Trott and Palmer equation.^[3]^ The exchange rate (k_ex_) was 534 ± 303 s^-1^. Experiments were recorded at 37.5 ºC at 700 MHz.


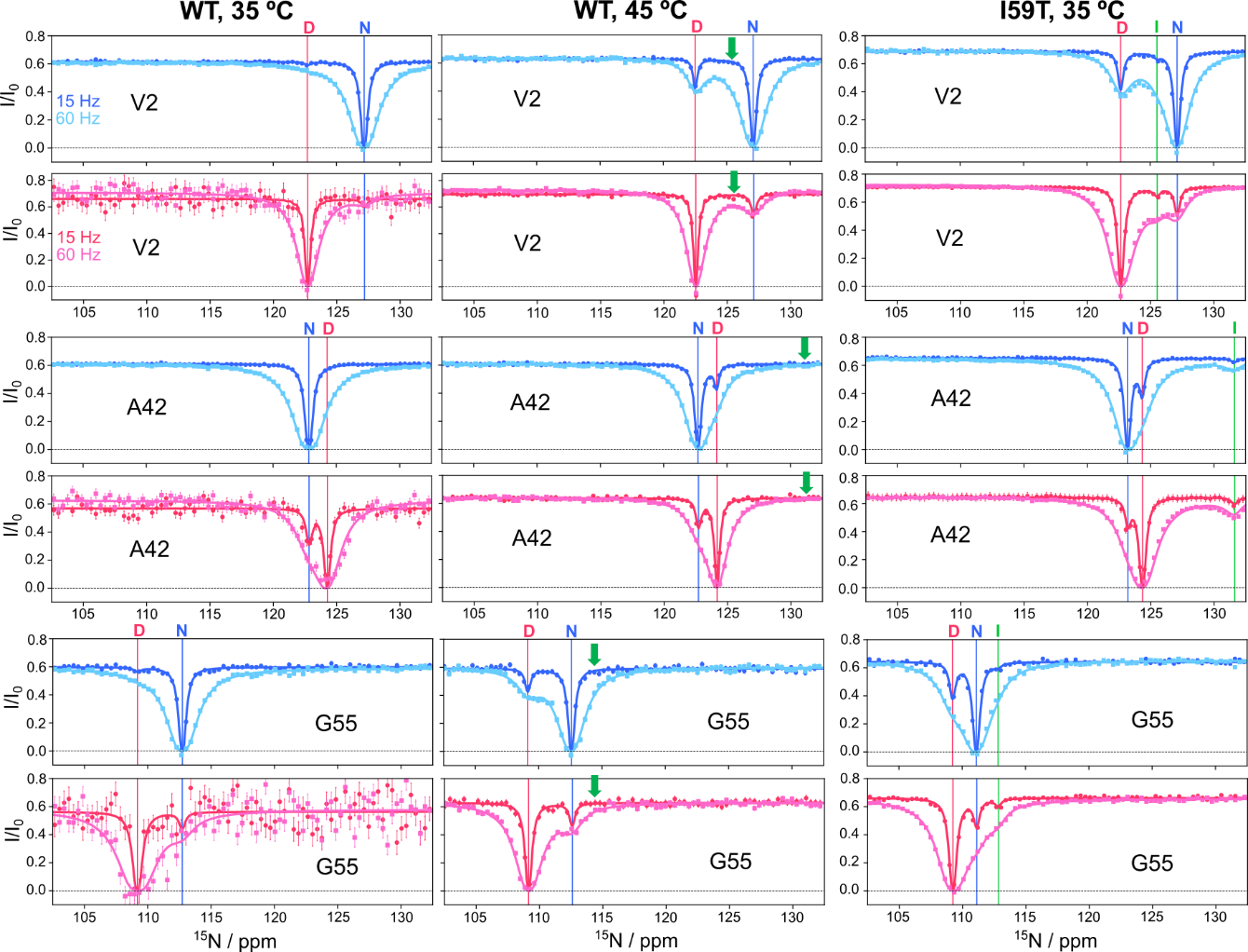


**Figure S6.** ^15^N CEST data for V2, A42 and G55 in the WT (35 and 45 ºC) and I59T (45 ºC). Red and Blue data points show the CEST data from the native and denatured peaks, respectively. Green arrows highlight the absence of intermediate state CEST dips in the WT data. All spectra were recorded at 800 MHz. Blue, red and green vertical lines indicate the chemical shift of the native, denatured and intermediate states, respectively.


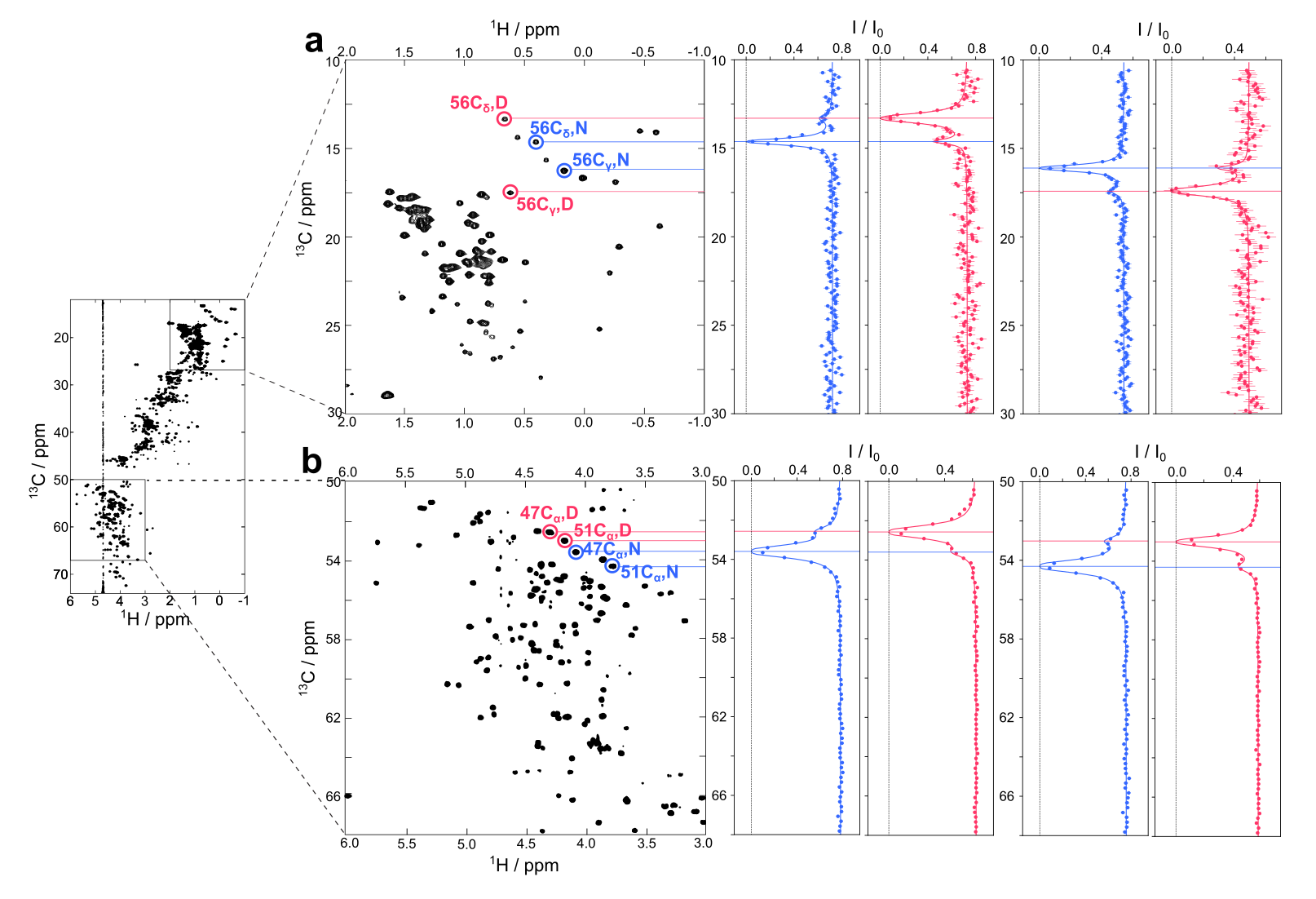


**Figure S7.** ^13^C CEST of I59T at 35 ºC. (a) ^13^C CEST on 56C_γ_ and 56C_δ_ recorded at 950 MHz. (b) ^13^C CEST on 47C_α_ and 51C_α_.


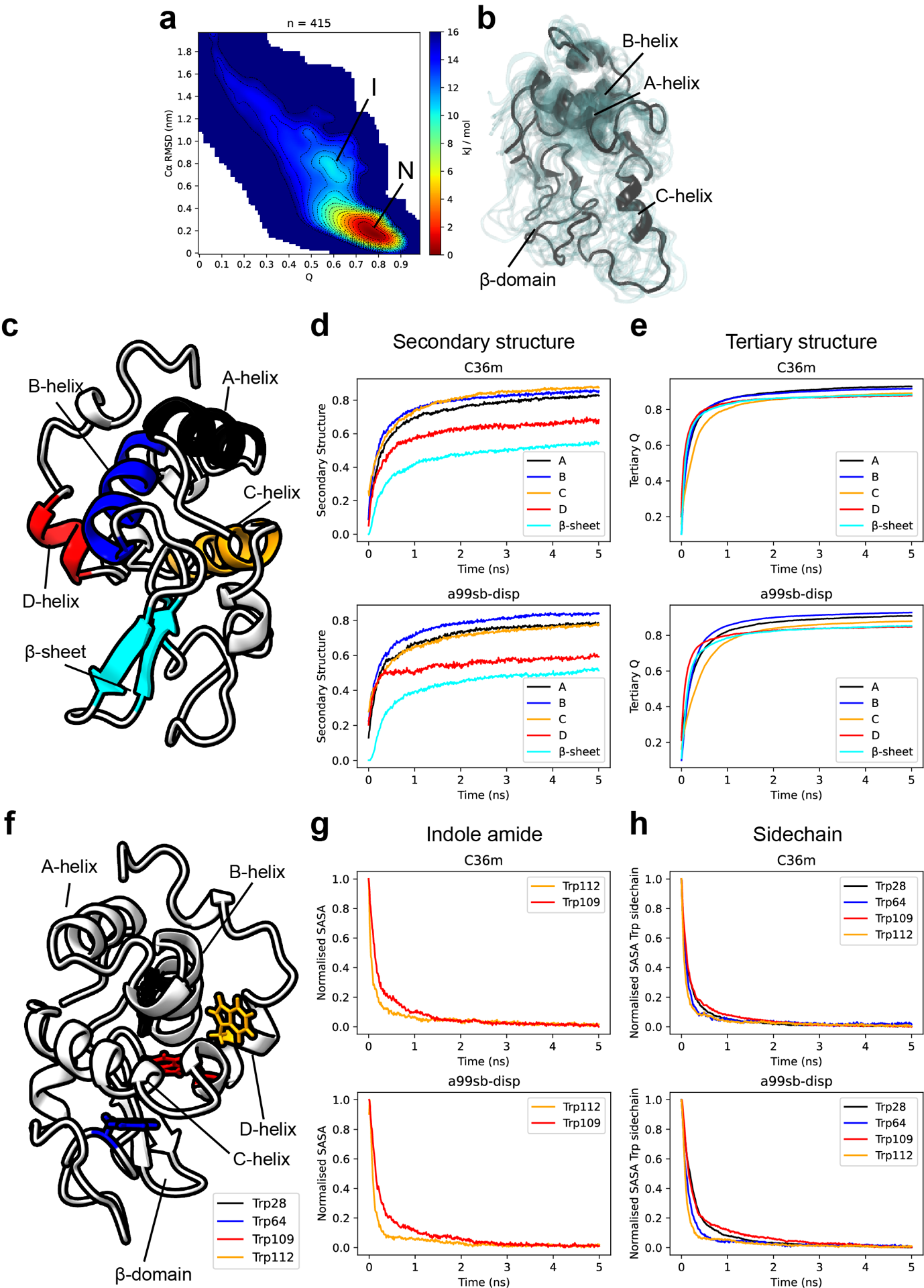


**Figure S8.** Analysis of the folding free energy landscape and pathway observed in rMD simulations. (a) Kinetic folding free energy landscape of HuL I59T calculated from all (n = 415) rMD trajectories that reach the native state with the C36m force field. The regions of the native state and metastable intermediate are annotated. (b) Representative ensemble of the folding intermediate with an unfolded C-helix and β-domain from panel a. With C36m, this state is only the second most populated cluster, in contrast to a99sb-disp (the most populated cluster obtained with C36m corresponds to an unfolded helix A, which is not supported by experiments). (c) Crystal structure (PDB 2MEH)^[4]^ annotated with the main secondary structure elements. (d) Average fraction of secondary structure formed in each secondary structure element as a function of time for all rMD trajectories that reached the native state. Secondary structure populations were calculated using DSSP (see Methods). (e) Average fraction tertiary contacts, Q (native contacts of each secondary structure element with the rest of the protein, see Methods), as a function of time for all rMD trajectories that reached the native state. (f) Crystal structure annotated with the four buried tryptophan residues. (g) Average time course of the tryptophan indole (atoms NE1 and HE1) solvent-accessibility group for the indicated residues from all rMD trajectories that reached the native state. The SASA was normalized by the minimum and maximum values observed to account for intrinsic differences between the two tryptophan residues in the native state. (h) SASA time courses for all four buried tryptophan sidechains.


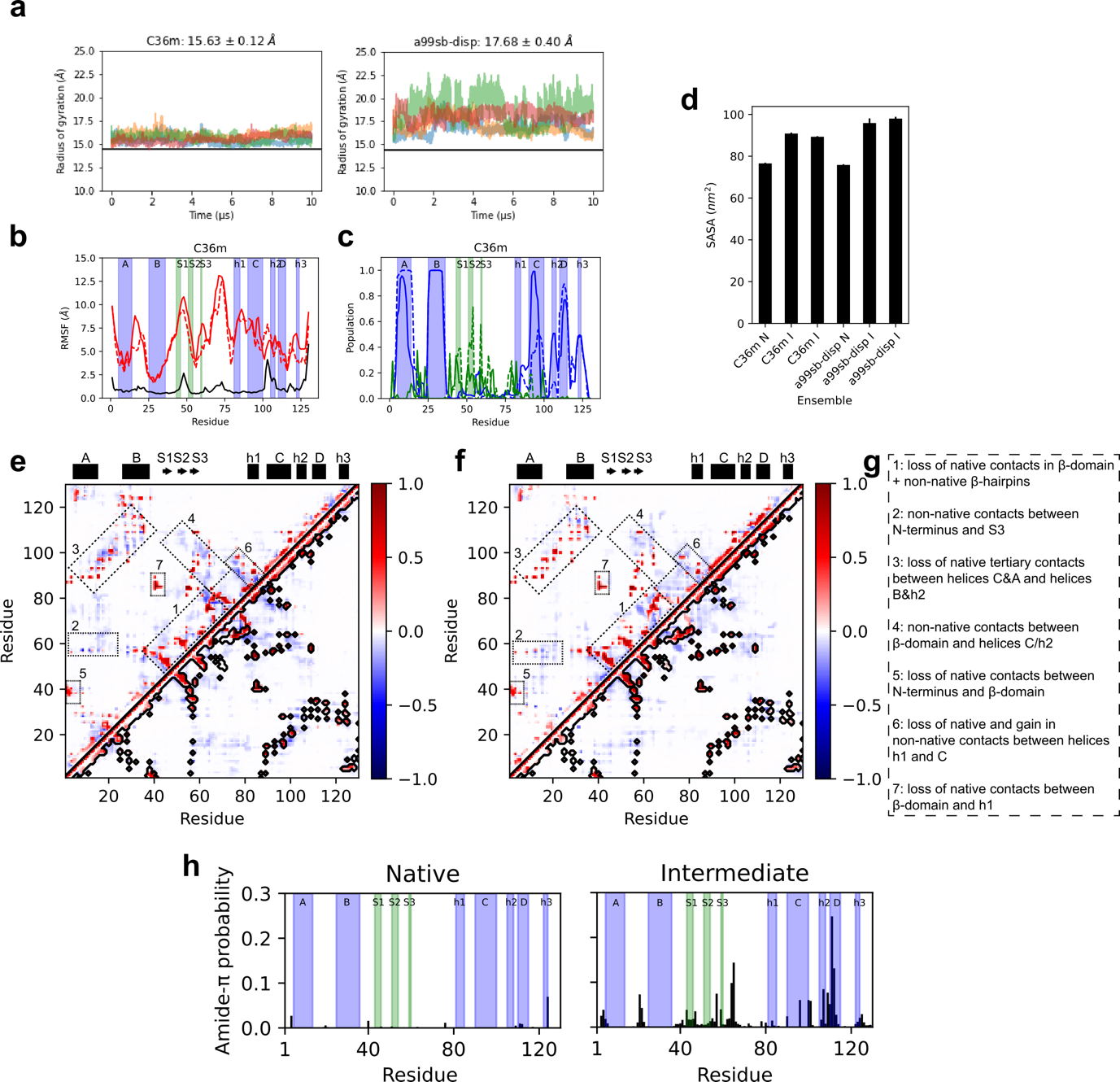


**Figure S9.** Conformational dynamics of the intermediate state ensemble sampled by unbiased MD simulations. (a) Stability of intermediate states in unbiased, long-timescale MD simulations assessed by fluctuations in the radius of gyration for both force fields. Each plot contains 8 x 10 μs trajectories generated from two starting structures (four simulations each). The horizontal line represents the radius of gyration obtained from the native state MD simulations (average ± standard error from 4 x 2.5 μs). (b) Backbone dynamics of the native (black) and intermediates state (red) calculated from the unbiased MD simulations for the C36m force field, quantified by the RMSF (Cα atoms). The solid and dashed red lines represent the RMSF calculated from 4 x 10 μs of unbiased MD simulations for each intermediate starting structure to assess the reproducibility of the structural ensemble. (c) Average secondary structure populations (α-helix – blue, β-sheet – green) of the intermediate state ensemble obtained with the C36m force field (solid and dashed line represent the 4 x 10 μs ensembles from two starting structures, respectively). (d) Average solvent-accessible surface area (SASA) of the native state (N, 4 x 2.5 μs) and intermediate state ensembles for both force fields (I, 4 x 10 μs). (e) Difference contact map of the intermediate state (native – intermediate) for the intermediate state ensemble obtained with the C36m force field. Contacts were defined when heavy atoms of two residues come within 5.0 Å of each other. Red represents the loss of native contacts and blue the gain in non-native contacts. The black contour is the contact map of the native state ensemble. (f) Difference contact map of the intermediate state obtained with the a99sb-disp force field. (g) Description of highlighted regions in the contact maps in panels e-f. (h) Ensemble-averaged probability of forming amide-π interactions in the native and intermediate state ensemble obtained with C36m.


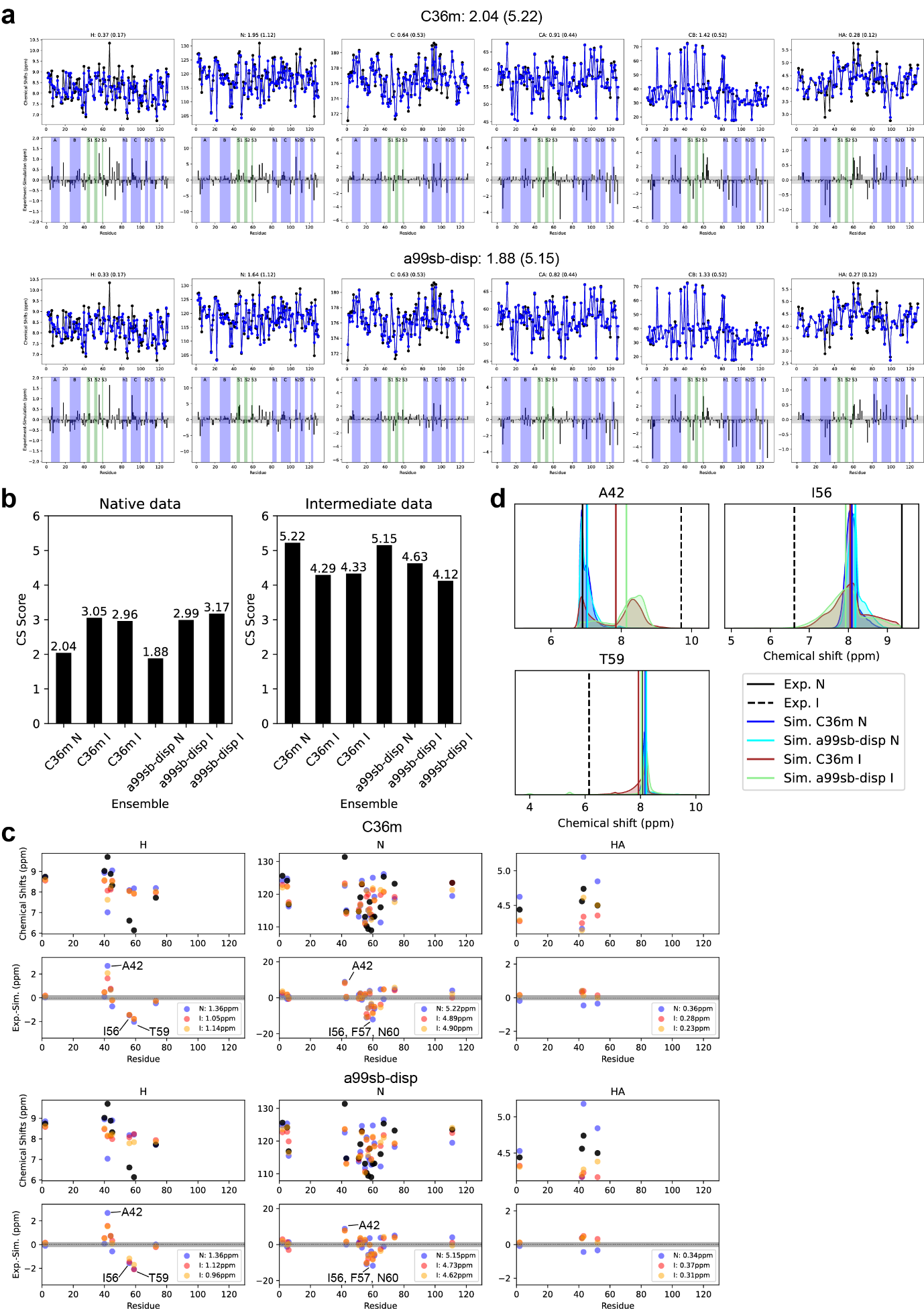


**Figure S10.** Analysis and comparison of back-calculated NMR chemical shifts with experiments. (a) Comparison of experimental the back-calculated backbone chemical shifts of the native state. The top row contains the experimental values (black) and ensemble-averaged values obtained by MD (4 x 2.5 μs). The bottom row shows the difference between the experimental and simulated values. The shaded area in grey represents the average error of the forward model (see Methods). For each force field, the average chemical shift score (see Methods) for the native and intermediate (in parentheses) state are shown. (b) Agreement between all MD ensembles and the experimental data for the native (left) and intermediate (right) state, quantified by the chemical shift score. (c) Comparison of experimental the back-calculated backbone chemical shifts of the intermediate state. For each force field, the top row shows the experimental (black) and simulated values, and the bottom row shows the difference of the simulated values from the experimental data (grey shaded areas highlight the average uncertainty of the forward model). Residues 42, 56, 57, and 59 exhibit the largest deviation from the experimental values. The average deviation for each nucleus is shown in the figure legend of the bottom row. (d) Probability distributions of the sampled amide proton chemical shifts by MD in the native and intermediate states. The experimental values are indicated with vertical lines in black and MD ensemble average are also shown in vertical lines.

**References**

[1] a) D. Sharma, K. Rajarathnam, *J. Biomol. NMR* **2000**, *18*, 165; b) O. A. Martin, M. E. Villegas, J. A. Vila, H. A. Scheraga, *J. Biomol. NMR* **2010**, *46* (3), 217.

[2] R. Wain, L. J. Smith, C. M. Dobson, *J. Mol. Biol.* **2005**, *351* (3), 662.

[3] O. Trott, A. G. Palmer III, *J. Magn. Resn.* **2002**, *154* (1), 157.

[4] J. Funahashi, K. Takano, Y. Yamagata, K. Yutani, *Protein Eng.* **1999**, *12* (10), 841.
